# From cracks to informed circularity: Mechanics-guided decisions via high-throughput in situ failure analysis of recycled plastics

**DOI:** 10.1126/sciadv.aeh0456

**Published:** 2026-07-23

**Authors:** Danqi Sun, Yiming Xu, Christos E. Athanasiou

**Affiliations:** ^1^ Daniel Guggenheim School of Aerospace Engineering, Georgia Institute of Technology, Atlanta, GA 30332, USA.; ^2^ Department of Electrical and Computer Engineering, University of Virginia, Charlottesville, VA 22903, USA.

## Abstract

Materials under real-world conditions are subjected to coupled mechanical and chemical stressors, yet current testing platforms cannot capture their combined effects on material behavior and failure, particularly when multiple specimens are required for robust measurements. This limitation is acute for recycled materials, whose performance cannot be inferred from a few conventional tests. Here, we introduce an in situ, high-throughput photoelasticity platform for multispecimen testing under controlled chemical conditions with full-field stress visualization. Using recycled polyethylene terephthalate (rPET) as a model system under simultaneous loading and varying pH, we reveal earlier onset of crack propagation and shorter lifetimes compared to virgin PET and quantify mechanochemical process zone expansion from ∼0.08 to ∼0.25 millimeters before failure. Extending these findings to landfill geotextiles, a growing application of rPET, we show that alkaline degradation erodes rPET environmental and economic advantages over virgin PET above pH 9. This integrated workflow, linking instrumentation to mechanistic insights to sustainability-informed decision-making, can accelerate the deployment of emerging, circular materials.

## Teaser

High-throughput testing under mechanical load and chemical exposure reveals recycled plastic failure and sustainability tradeoffs.

## INTRODUCTION

Materials in real environments rarely experience a single, isolated stressor. Instead, they operate under multifactor, fluctuating, and often harsh conditions, from sustained mechanical loads (*1*–*3*), chemical exposures to temperature cycles (*4*–*6*), humidity (*7*–*9*), or radiation (*10*). These coupled stimuli dictate service life and failure pathways, shaping the socio-environmental and economic impacts of the infrastructures and products that rely on them (*11*). Addressing pressing global challenges, like plastic pollution (*12*–*14*), increasingly depends on experimental platforms capable of investigating materials under such complex, realistic conditions, ideally with multiple specimens tested simultaneously to capture variability and accelerate discovery (*11*). Yet, existing testing approaches fall short in this regard. Traditional testing machines offer robust mechanical control but limited environmental versatility (*15*, *16*), commercially available environmental chambers allow for temperature or pH control but lack in situ characterization (*17*, *18*), and high-throughput platforms prioritize specimen multiplicity at the expense of environmental complexity (*19*). They are also unable to quantify stress fields or capture slow mechanochemical fracture phenomena. As a result, there is a testing trade-off between specimen number and environmental conditions, hence, critical mechanistic data needed to predict lifetime and ensure reliable deployment under diverse environments remain largely missing.

These gaps are especially limiting for emerging materials whose performance cannot be inferred from conventional single-specimen, single-condition tests (*20*). For example, recycled polymers, bio-based plastics, and vitrimers often exhibit heterogeneous microstructures that are sensitive to chemical environments and highly susceptible to environmentally assisted degradation (*21*–*24*). Although specialized systems exist for fracture testing in corrosive media for these materials, they are almost exclusively single-specimen tools; conversely, automated micromechanical and indentation platforms enable high throughput testing but operate in benign laboratory conditions (table S1) (*25*, *26*). Consequently, the mechanistic pathways governing long-term degradation for such materials, particularly under chemically aggressive environments, are poorly understood.

This knowledge gap is notable in the context of global plastic pollution and the urgent need to expand circular materials infrastructures. Polyethylene terephthalate (PET) is among the most widely used, disposed, and nominally recycled polymers worldwide (*27*, *28*). Although recycled PET (rPET) is commonly used in packaging and textiles, there is growing interest in deploying it in more demanding, longer-lived applications (*29*). Among these, geotextiles and landfill liners represent a rapidly expanding use of rPET. These systems experience alkaline environments, often at pH ≥ 9, due to soil chemistry and evolving landfill leachates (*30*–*32*). Under these conditions, PET undergoes hydroxide-catalyzed chain scission, diffusional softening, and unconventional crack growth (*33*, *34*). Nearly all existing assessments for rPET rely on bulk tensile strength, accelerated aging protocols, or creep rupture tests in simplified environments (*35*, *36*). Mechanochemical process zones, crack tip chemistry, ductile-to-brittle transitions, and environmentally assisted crack growth in rPET have never been systematically quantified, leaving critical uncertainties in decision making about when this material can truly offer environmental and economic benefits in demanding applications.

**Fig. 1.**
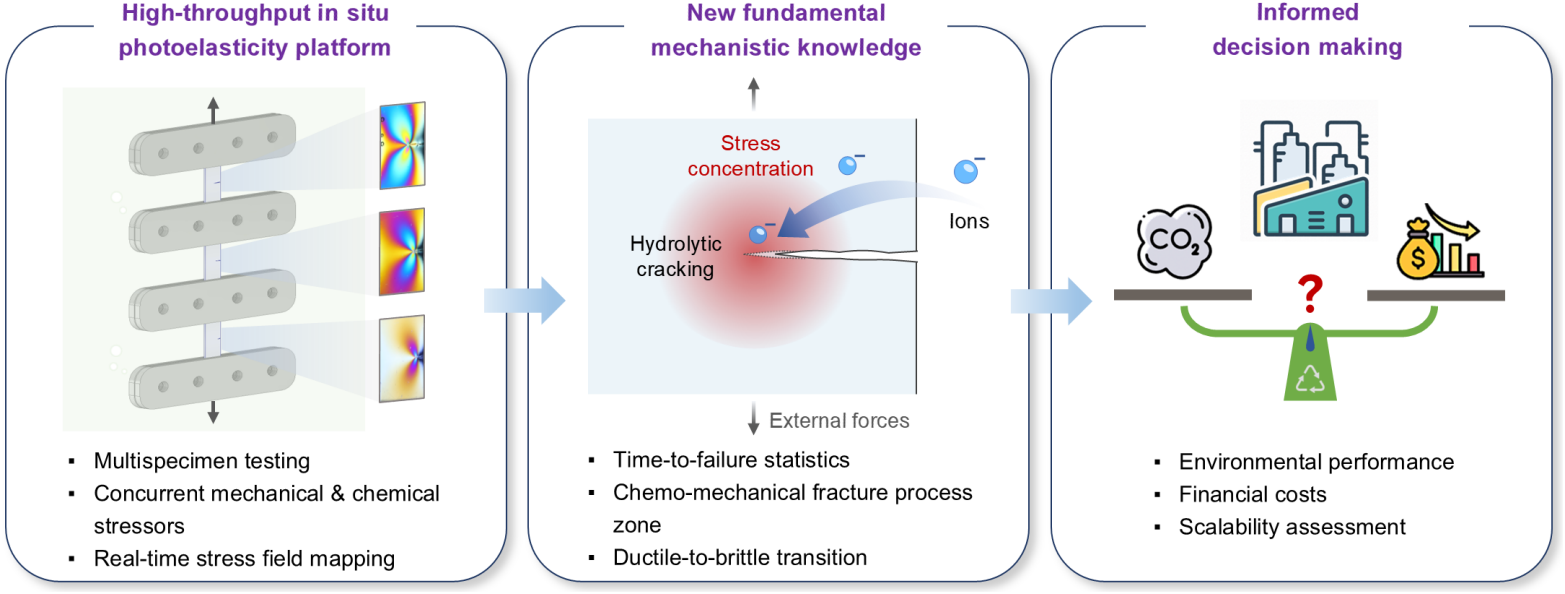
Workflow: From high-throughput testing to sustainable engineering decision-making. The high-throughput in situ photoelasticity platform accelerates data acquisition while directly capturing the evolution of stress fields, providing new fundamental mechanistic insights into ESC of recycled plastics, and ultimately guiding environmentally and economically informed engineering decisions. Icons in right were adapted from free resources available at Flaticon (https://flaticon.com), used in accordance with Flaticon’s Free License with attribution (made by authors: Three musketeers, Freepik, Uniconlabs).

To address these gaps, we developed a mechanical testing platform that enables: (i) multispecimen testing, (ii) long-term testing under controlled chemical conditions, and (iii) in situ full-field stress visualization through photoelasticity. We applied this platform to PET and rPET to demonstrate an end-to-end workflow: from high-throughput testing to mechanistic characterization to sustainability-driven decision guidance (Fig. 1). We tested virgin PET and rPET (50% recycled content) specimens across pH 7 to 13, where the applied load and initial crack length produced energy release rates of 0.002 to 5 kJ m^−2^. Across this range, rPET specimens consistently exhibited faster crack growth and shorter lifetimes than virgin PET; under the same pH conditions, rPET is more prone to crack propagation, with an environmental stress cracking (ESC) resistance that is 50% lower than that of virgin PET. In situ photoelasticity further showed that the mechanochemical process zone in rPET expanded with increasing energy release rate and with time before failure. We then integrated these mechanistic insights into an environmental and economic assessment of rPET geotextile landfill liners, illustrating how such data can directly inform real-world decisions. Our analysis shows that geotextiles made from 50% rPET blends are neither environmentally nor economically favorable once the surrounding environment exceeds pH 9, a previously unrecognized threshold with implications for landfill design, material selection, and circularity policies. Collectively, this work establishes a workflow capable of generating the mechanistic evidence required to evaluate emerging materials and guide their deployment across diverse applications.

## RESULTS

### In situ high-throughput photoelasticity testing platform

Plastics often undergo mechanical degradation during prolonged exposure to chemically aggressive environments, but long-term ESC experiments are time intensive and data sparse (*37*–*40*). To address this limitation, we developed an in situ, high-throughput photoelasticity platform capable of evaluating the ESC behavior of polymer films under controlled chemical conditions.

**Fig. 2.**
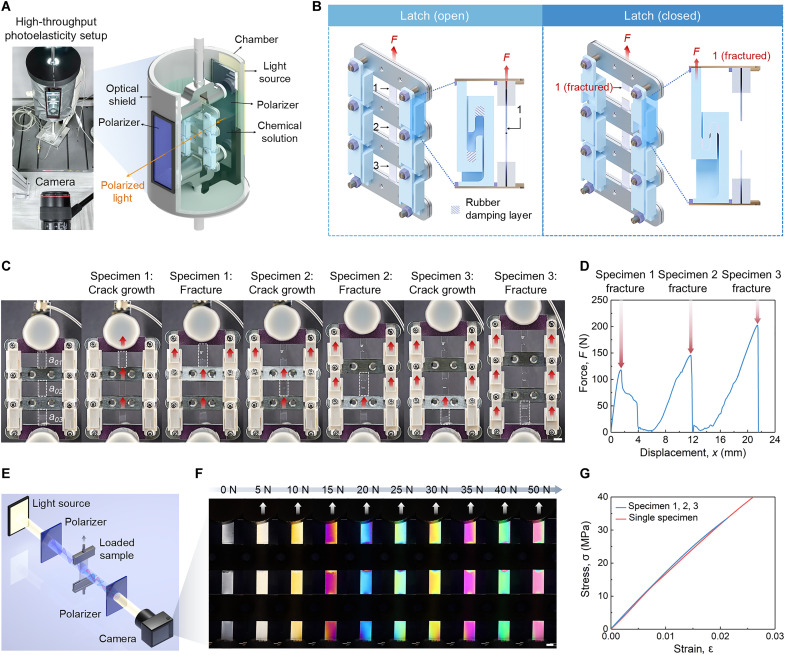
In situ high-throughput photoelasticity testing platform. (**A**) Photograph of the testing platform and internal schematic of the environmental chamber. (**B**) Design of the high-throughput testing framework. Multiple specimens are simultaneously loaded with the applied load, *F*, with the design ensuring that their mechanical responses remain independent. (**C**) Loading in air of three specimens with different initial crack lengths (*a*_01_ > *a*_02_ > *a*_03_) mounted in the high-throughput framework, showing sequential failure process of the three specimens. Scale bar, 10 mm. (**D**) The corresponding force-displacement (*F*-*x*) curve of high-throughput fracture test. (**E**) Illustration of the RGB photoelasticity. (**F**) Loading process of three identical specimens mounted in the high-throughput framework, showing identical birefringence during loading. Scale bar, 5 mm. (**G**) Stress-strain curves of the three specimens, compared with the curve from a single specimen tested individually.

A commercial environmental chamber was modified and equipped with a white light source, two polarizers, and an optical shield, enabling real-time acquisition of photoelasticity images during loading of specimens immersed in aqueous solution (Fig. 2A). In parallel, we designed a high-throughput testing framework that allows multiple specimens to be tested simultaneously under the same load (Fig. 2B). Three single-edge-notched specimens are mounted in series, each subjected to the same applied force *F*. Before failure, each specimen’s latch remains open, ensuring that the specimen carries the full load. When a specimen fails, its corresponding latch closes instantaneously, maintaining continuous loading on the remaining specimens. A built-in rubber damping layer mitigates the transient impact associated with latch closure, ensuring that the mechanical responses of the remaining specimens remain independent.

By using specimens with different initial crack lengths (here *a*_01_ = 3.1 mm, *a*_02_ = 1.9 mm, and *a*_03_ = 0.8 mm), the framework yields three complete stress-strain curves within a single tensile test. As expected, the specimen with the longest crack fails first, followed by sequential failure of the medium and short-crack samples (Fig. 2C and movie S1). Each failure corresponds to a peak in the load-displacement curve (Fig. 2D). Although the overall load momentarily drops to zero during latch closure, the independently tracked deformation of each specimen shows that their individual stress-strain responses (fig. S1) remain unaffected, confirming that transient unloading effects are negligible. The high-throughput testing framework enables the acquisition of three sets of stress-strain (σ-ε) responses within a single tensile test.

To obtain in situ mechanistic information during long-term loading, we integrated a photoelasticity module into the platform. Photoelasticity quantifies stress-induced birefringence as specimens are loaded within a polarized light field (Fig. 2E) (*41*, *42*). In our implementation, red-green-blue (RGB) image acquisition captures wavelength-dependent birefringence across three channels simultaneously, enabling reliable reconstruction of principal stress differences and revealing subtle crack tip stress redistribution. When uniaxial tension is applied to three PET specimens of identical geometry, all three samples exhibit identical RGB birefringence patterns during loading (Fig. 2F), confirming that each specimen in the high-throughput framework experiences the same applied load *F*. Because the strain of each specimen is also identical, the resulting stress-strain (σ-ε) curves match well with individually tested sample (Fig. 2G). These results demonstrate that the in situ high-throughput photoelasticity platform can simultaneously load multiple specimens while maintaining independent mechanical responses, enabling the acquisition of multiple mechanical datasets and full-field stress information within a single experiment.

### Hydrolytic mechanical degradation

**Fig. 3.**
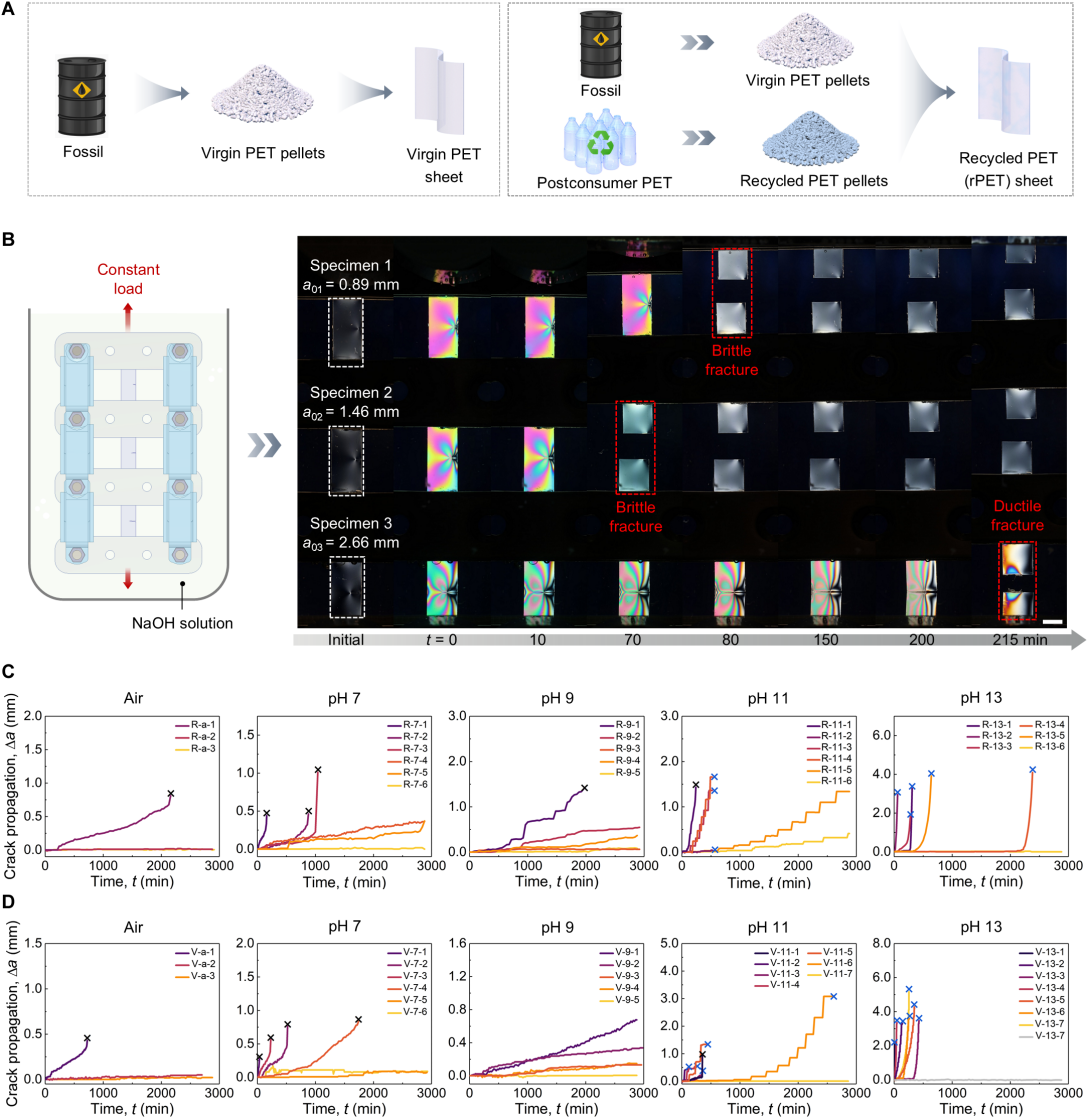
ESC tests of recycled rPET. (**A**) Manufacturing of virgin PET and rPET (50% recycled content). (**B**) rPET specimens subjected to a constant load using the in situ high-throughput photoelasticity testing framework in an alkaline solution. The images show the ductile-to-brittle transition of three rPET specimens with different initial crack lengths with an applied load of 65 N under pH 11 condition. Scale bar, 5 mm. High-throughput measurements of crack propagation Δ*a* as a function of time *t*, for (**C**) rPET and (**D**) virgin PET specimens, tested under five environmental conditions: air and NaOH solutions of pH = 7, 9, 11, and 13. Black × markers denote ductile fracture, and blue × markers denote brittle fracture.

We used this platform to investigate the hydrolysis-driven mechanical degradation of PET and rPET under alkaline conditions. A commercial postconsumer rPET blend (50% recycled content; 50% virgin polymer) was compared with virgin PET to assess how recycled content affects material’s long-term durability (Fig. 3A). Fourier-transform infrared spectroscopy confirms that the chemical bonds and functional groups of the two materials are essentially identical (fig. S2). Differential scanning calorimetry (DSC) reveals crystallinities of 13.4 and 11.2% for the rPET blend and virgin PET, respectively. The DSC scan of rPET exhibits an indistinct glass transition (*T*_g_) and broader melting (*T*_m_) and crystallization (*T*_c_) peaks, indicating a less ordered molecular-chain structure with a higher fraction of short chains (fig. S3). Under uniaxial tension, rPET exhibits a slightly higher elastic modulus (*E* = 1994 ± 108 MPa) and yield stress (σ*_y_* = 55 ± 4 MPa) than virgin PET (*E* = 1673 ± 92 MPa, σ*_y_* = 50 ± 3 MPa), but its fracture energy (*w*_e_ = 8260 J m^−2^) is ∼3× lower than that of virgin PET (*w*_e_ = 22630 J m^−2^), consistent with shorter chain lengths and reduced entanglement density in rPET (figs. S4 and S5).

Hydrolytic degradation of PET is a long-term, multiphysics process governed by the interplay of hydroxide-driven chain scission, solvent diffusion, and the evolving stress gradients near cracks (*43*, *44*). To isolate chemical erosion from mechanically driven effects, specimens were first hydrolyzed under stress-free conditions to eliminate the influence of residual stresses and subsequently subjected to mechanical testing (fig. S6). Uniaxial tension tests show that 5 days of hydrolysis lead to only slight reductions in *E* and σ*_y_* for virgin PET, whereas the rPET blend degrades more noticeably, with these properties decreasing to 82 and 87% of their initial values, respectively (figs. S7 and S8). Stress relaxation tests reveal progressively stronger viscoelastic decay with hydrolysis time for both materials. On the basis of prior work (*45*), we attribute this change in behavior in water and ion diffusion that disrupts noncovalent interactions and promote chain mobility (figs. S10 and S11). Stress relaxation saturates after ∼3 days, and samples hydrolyzed for 5 days were selected for ESC experiments.

When cracks or defects are present, stress gradients further accelerate hydrolytic degradation. The testing platform enables rapid acquisition of ESC data and direct visualization of crack tip stress evolution across different chemical environments. Three specimens with different initial crack lengths were subjected to a constant force *F* for 2 days or until all specimens fractured, in air and alkaline solutions (pH 7, 9, 11, and 13). The differing initial crack lengths generated distinct initial applied energy release rates *G*_0_ and thus different stress gradients within each specimen (Fig. 3B and movie S2). Under combined hydrolysis and loading, cracks propagated gradually, and the crack propagation Δ*a*(*t*) was independently measured for each specimen (Materials and Methods).

The rPET blend exhibits ductile fracture at pH 7 and 9 (figs. S12 and S13) and brittle fracture under pH 11 and 13 (Fig. 3B and fig. S14), consistent with previously reported hydrolytic cracking behavior of virgin PET (*33*). The high-throughput testing framework captures this ductile-to-brittle transition within a single experiment (Fig. 3B). At pH 11, specimens 1 and 2 (with smaller initial cracks and lower *G*_0_) show limited crack growth under a 65 N load before undergoing sudden brittle fracture at 70 and 80 min. In contrast, specimen 3, with the largest precrack and highest *G*_0_, undergoes ductile fracture at ∼215 min, which is attributed to the more extended plastic zone that provides crack tip shielding that delays hydrolysis-induced embrittlement.

Using the high-throughput platform, we tested more than 60 specimens, reducing the experimental time by ∼60% relative to single-specimen testing. This enabled us to obtain Δ*a*(*t*) curves for both rPET (Fig. 3C) and virgin PET (Fig. 3D) across five environmental conditions, providing the basis for quantitative analysis and modeling of hydrolytic crack growth. Representative single-specimen and multiple-specimen ESC tests under comparable loading and environmental conditions showed similar photoelastic evolution during crack growth (fig. S15), further supporting the reliability of the high-throughput configuration. The Δ*a*(*t*) curves are labeled by specimen index, with the corresponding initial crack length, applied stress, and initial energy release rate listed in tables S2 and S3.

### Hydrolytic crack growth behavior

**Fig. 4.**
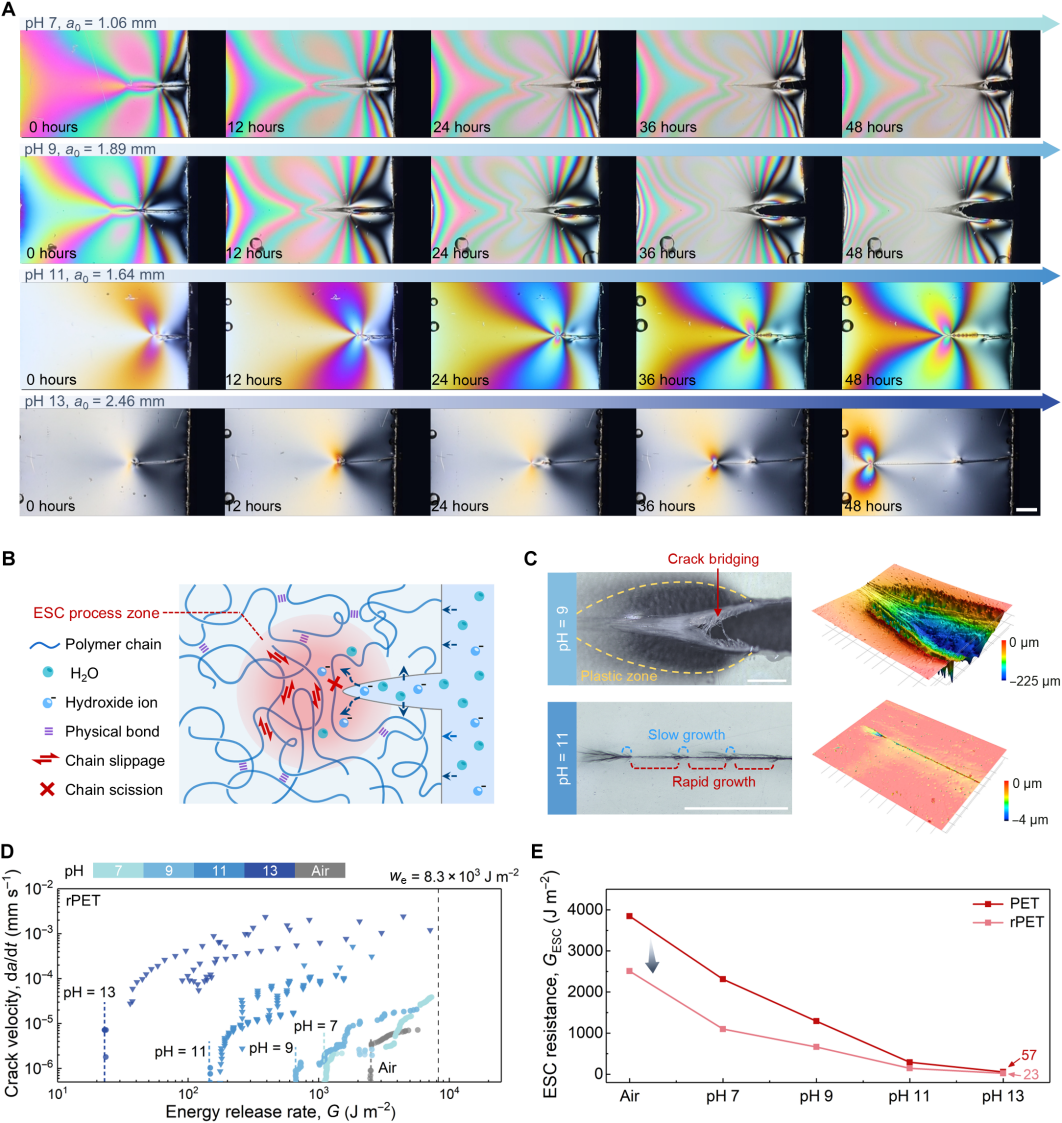
ESC behavior and underlying mechanisms of rPET in alkaline solutions. (**A**) rPET specimens exhibit ductile fracture under pH 7 and pH 9, characterized by pronounced plastic yielding and crazing, whereas under pH 11 and pH 13, rPET specimens undergo brittle fracture with cracks propagating along a straight path. Scale bar, 1 mm. (**B**) Schematic illustration of the ESC mechanism of rPET in alkaline solutions. Stress concentration facilitates the penetration of water molecules and hydroxide ions into the crack tip, forming an ESC process zone that promotes chain slippage and chain scission. (**C**) 2D and 3D fractography characterizing ductile fracture (pH 9) and brittle fracture (pH 11). Scale bar, 500 μm. (**D**) Relationship between crack velocity d*a*/d*t* and the applied energy release rate *G* of rPET under different environmental conditions. The fracture toughness *w*_e_ of rPET under monotonic loading is 8300 J m^−2^. (**E**) Comparison of ESC resistance *G*_ESC_ for rPET and PET under various environmental conditions.

The high-throughput tests not only provide a comprehensive Δ*a*-*t* dataset but also enable continuous in situ observation of all specimens, allowing detailed characterization of hydrolytic crack growth mechanisms. As shown in Fig. 4A, the rPET blend requires relatively high *G*_0_ to initiate crack growth in mildly alkaline environments (pH 7 to 9), ultimately failing by ductile fracture. Photoelasticity reveals localized plastic deformation at the crack tip. Under sustained applied load, specimen undergoes creep, and steady-state crack growth proceeds through craze formation and coalescence (*46*), while the plastic zone remains approximately constant in size. In this regime, crack advance is driven primarily by mechanical stress, whereas hydrolysis enhances chain mobility by breaking physical interactions and promoting chain slippage (Fig. 4B). Two-dimensional (2D) fractography shows that a plastic zone exceeding ∼1.5 mm under ductile fracture at pH 9. Plastic flow and chain orientation within the amorphous phase lead to microscopic void nucleation and fibril formation (*47*), producing crack bridging with fibrils ∼100 to 350 μm in length (Fig. 4C).

By contrast, in strongly alkaline environments (pH ≥ 11), aggressive hydrolysis dominates crack growth behavior and enables crack propagation at applied *G*_0_ values an order of magnitude lower than in mildly alkaline conditions. Photoelasticity reveals pronounced stress concentration at the crack tip (Fig. 4A). These steep stress gradients accelerate the penetration and transport of water molecules and hydroxide ions into the crack tip region, forming a mechanochemical ESC process zone (Fig. 4B). Base-catalyzed hydrolysis of ester bonds induces localized embrittlement at the crack tip, lowers the activation energy barrier for chain scission, and triggers crack advance. As new crack surfaces form, penetration resumes, generating repeated cycles of localized embrittlement. This mechanochemically coupled fracture process produces the characteristic “slow-fast-slow” stepwise crack growth pattern, as evidenced by the Δ*a*(*t*) curves (Fig. 3C) and 2D fractography (Fig. 4C).

3D fractography further distinguishes these regimes. Brittle fracture produces minimal plastic deformation and a damage depth of only ∼4 μm, whereas ductile fracture generates a large damage zone of ∼225 μm ahead of the crack tip.

During ESC, the hydrolytic crack growth rate d*a*/d*t* depends on both the environmental conditions and the applied energy release rate *G*. For a given *G*, stronger alkaline environments induce faster crack growth (Fig. 4D and fig. S16). When *G* is very low, crack propagation is negligible. We define the ESC resistance *G*_ESC_ as the critical energy release rate at d*a*/d*t* = 5 × 10^−7^ mm s^−1^, above which the crack enters slow propagation stage.

On the basis of the dataset obtained from the high-throughput tests, we developed a kinetic model to describe the hydrolytic crack growth of PET governed by mechanochemical coupling (see Materials and Methods) (*48*, *49*). In the slow propagation regime, the d*a*/d*t*-*G* relationship is expressed as

$$\text{ln}\left(da/dt\right)=\text{ln}\left(\nu \right)+\alpha \sqrt{G}$$ (1)

where ν is the intrinsic chemical reacting rate, and α describes the effect of mechanical stress on lowering the activation energy barrier. As shown in fig. S17, Eq. 1 captures the hydrolytic crack growth behavior of both rPET and virgin PET under alkaline conditions, although the rPET data exhibit some scatter. Table S4 shows that ν increases with increasing pH, while α exhibits negligible variation with environmental conditions but is consistently higher for the rPET blend than for virgin PET, indicating that larger defect population in rPET leads to a larger activation volume.

As pH increases, both rPET and virgin PET experience a pronounced acceleration of hydrolytic cracking, with their *G*_ESC_ decreasing by approximately two orders of magnitude. Although the *E* and σ*_y_* of rPET are comparable to those of virgin PET, the *G*_ESC_ of rPET is ∼50% lower across all testing environments (Fig. 4E).

Together, the thermal (slightly higher crystallinity and broadened transitions), mechanical (similar *E* and σ*_y_* but much lower *w*_e_), and kinetic data (∼50% lower *G*_ESC_ and higher α) indicate that rPET has shorter and more heterogeneous chains. These features lower the barrier for stress-assisted hydrolysis, explaining its reduced ESC resistance and increased vulnerability to hydrolytic cracking in aggressive alkaline environments.

### Stress field evolution during hydrolytic cracking

**Fig. 5.**
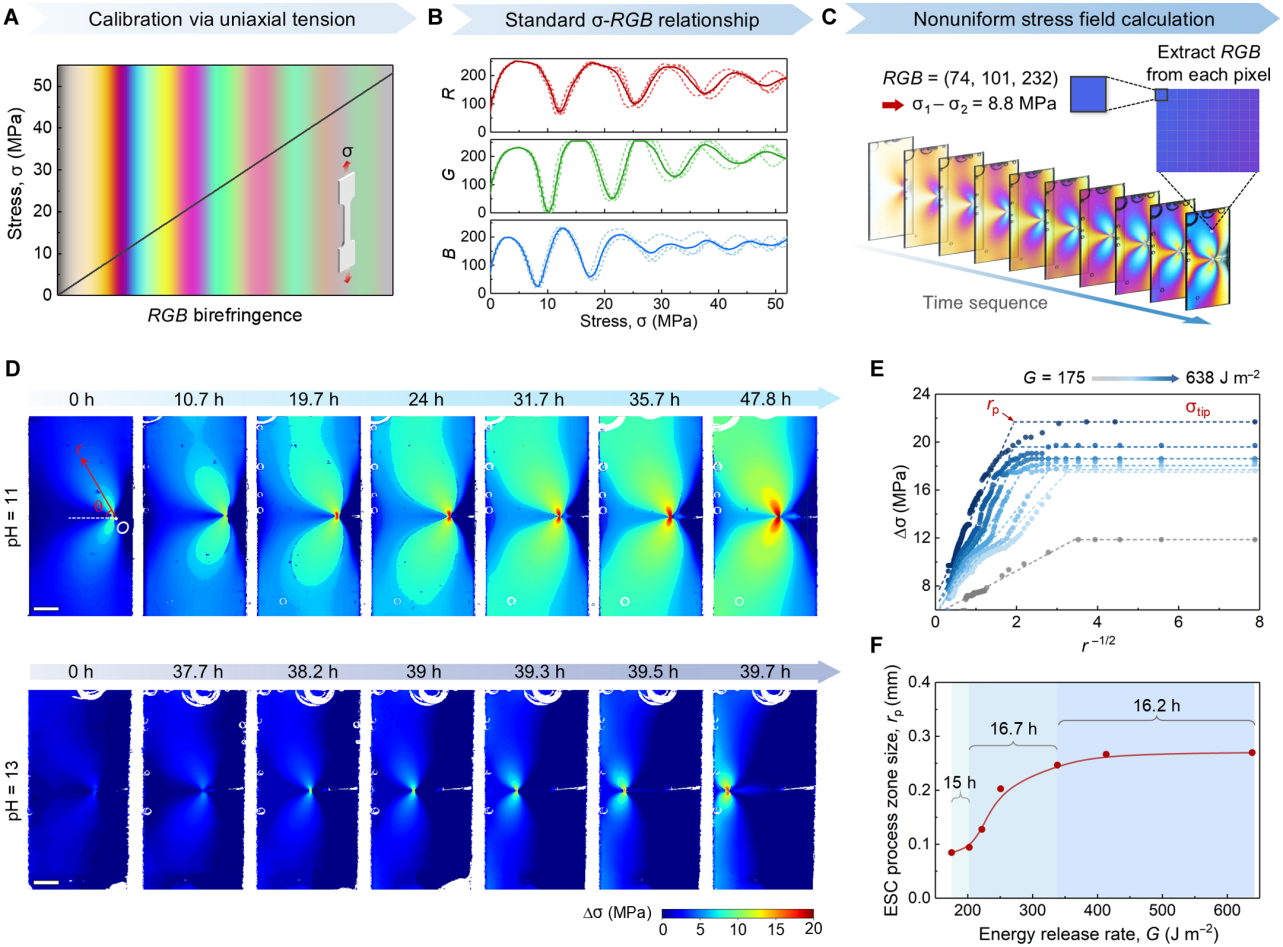
Stress field evolution of rPET during ESC measured by photoelasticity. (**A** to **C**) Workflow of the photoelasticity measurement for determining the stress field evolution: (A) uniaxial tensile calibration and (B) construction of the standard relationship between stress σ and birefringence (*R*, *G*, and *B*). (C) Pixel-wise mapping of the nonuniform stress field from birefringence images. (**D**) Stress field evolution during crack propagation under pH 11 and pH 13 conditions. A polar coordinate system (*O*, *r*, and θ) is introduced with *O* located at the crack tip. Scale bars, 2 mm. (**E**) Relationship between the stress difference Δσ and *r*^1/2^ at θ = 60° under pH 11. The applied energy release rate *G* increases from 175 to 638 J m^−2^. (**F**) Evolution of the ESC process zone size *r*_p_ with *G* under pH 11. The ESC process zone enlarges slowly [15 hours (h)], then expands rapidly to a quasi-steady state (16.7 hours), and lastly grows until complete failure (16.2 hours).

The photoelastic module integrated in the testing platform enables in situ measurement of the stress field evolution during hydrolytic cracking. To map the stress field from photoelastic images, we first calibrated the stress-optical relationship of rPET film. Under uniaxial tension, the specimen experiences a uniform stress σ, which can be correlated with the measured birefringence (Fig. 5A) (*50*). Hydrolytic erosion experiments show that the stress-optical relationship of both rPET and virgin PET evolves and shifts with erosion time under different alkaline conditions. Owing to the small film thickness, the stress-optical relationships stabilize after about 1 day (fig. S9). In computing the stress fields during hydrolytic cracking, we used the stabilized stress-optical relationships measured after 5 days of hydrolytic erosion. The birefringence pattern is decomposed into the three *RGB* channels for image processing, each of which is individually correlated with σ to obtain a calibrated σ-*RGB* relationship (Fig. 5B). For nonuniform stress distribution, the *RGB* values of each pixel are converted to the principal stress difference, Δσ = σ_1_ − σ_2_, using this calibration. Iterating over all pixels yields the stress field at a given time, and repeating this for each frame produces the full stress field evolution (Fig. 5C). Small bubbles can occasionally appear near specimen edges during long-term experiments because of trapped or released dissolved air. These regions are identified and excluded during image processing, with limited influence on the quantitative analysis of crack tip stress evolution.

This approach provides in situ visualization of stress field evolution during ESC in aggressive environments (Fig. 5D). Immediately after load *F* is applied, the amplitude of the stresses near the crack tip exhibits modest values. As hydrolysis progresses, the local ester bond scission reduces the load-bearing capacity of the zone around the crack tip, leading to progressive crack advance and eventual catastrophic failure. Defining a polar coordinate system (*O*, *r*, and θ) centered at the crack tip, the stress field at θ = 60° is evaluated through the relationship between Δσ and *r*^1/2^ (Fig. 5E and fig. S18A). The near-tip region can be divided into a G-annulus governed by *G* and an adjacent ESC process zone. Within the G-annulus, Δσ varies linearly with *r*^1/2^, with the slope increasing as *G* rises. At *r* = *r*_p_, Δσ reaches a saturated value σ_tip_ and becomes independent of *r*. We define *r*_p_ as the scale of ESC process zone, within which hydrolysis-induced localized embrittlement enables crack growth at stresses far below σ*_y_*. Under pH 13, σ_tip_ is lower than that at pH 11, indicating more severe local embrittlement in the stronger alkaline environment (fig. S18A).

During hydrolytic cracking, crack propagation increases *G* according to Eq. 2, driving the evolution of *r*_p_ (Fig. 5F and fig. S18B). At pH 11, *r*_p_ is initially small (*r*_p_ = 0.08 mm) and increases slowly at low *G*. After about 15 hours of hydrolysis, crack growth initiates, and the enhanced penetration and diffusion driven by rising *G* cause *r*_p_ to expand rapidly to ∼0.25 mm within 16.7 hours. As *G* continues to increase, the rate of penetration and diffusion can no longer keep pace with the generation of new crack surfaces, and *r*_p_ approaches a quasi-steady value of 0.27 mm (fig. S18C). At pH 13, *r*_p_ is initially 0.09 mm at *G* = 80 J m^−2^ and continues to increase with *G*, reaching 0.52 mm without achieving equilibrium before the final fracture (fig. S18D). The stress field evolution measured by photoelasticity shows that hydrolytic crack growth is governed by a mechanochemically driven ESC process zone at the crack tip, where the local stress saturates at a pH-dependent σ_tip_, while the zone size *r*_p_ is jointly controlled by *G*, diffusion, and hydrolysis.

### Case study: Landfill liner sustainability performance

**Fig. 6.**
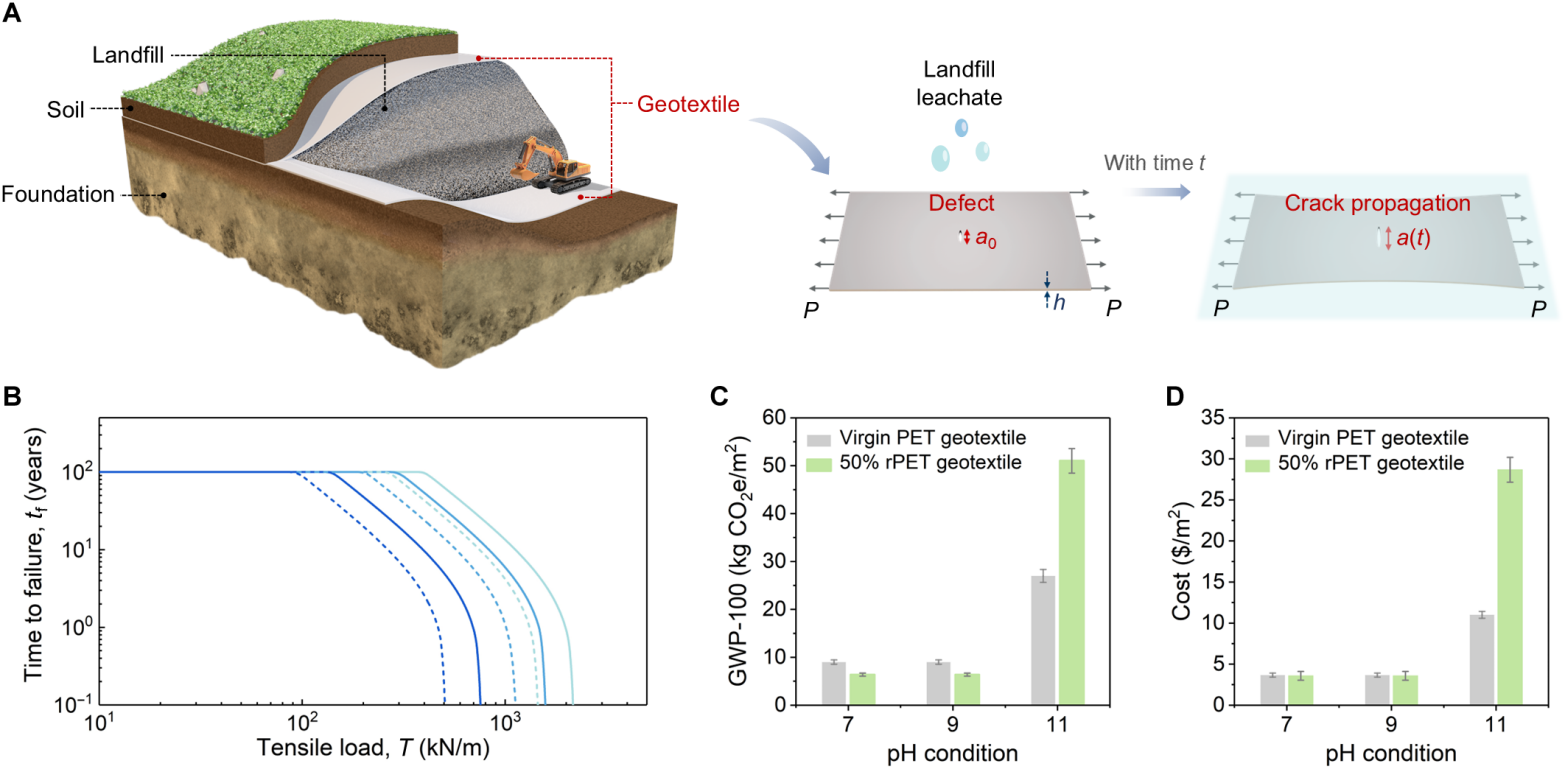
Case study: sustainability performance of landfill liners. (**A**) PET geotextile film used as a landfill liner. Landfill leachate accelerates ESC in a geotextile film of thickness *h*, causing defects to grow from *a*_0_ to *a*(*t*) over time *t* under a constant applied tensile load *P*. (**B**) Relationship between the time to failure *t*_f_ and *P*, under different alkaline environments (pH 7, 9, and 11). Solid lines represent virgin PET geotextile film, and dashed lines represent rPET blend geotextile film. (**C**) Environmental performance was quantified by comparing the 100-year global warming potentials (GWP-100) of virgin PET and rPET blend geotextile films under different conditions. (**D**) Cost comparison of PET and rPET blend geotextile films under different pH conditions.

Geotextiles play a critical role in landfill engineering by providing separation, reinforcement, and leachate containment and must maintain long-term structural integrity under continuous exposure to landfill leachate (Fig. 6A) (*30*, *31*). PET, widely used in geotextile manufacturing and increasingly sourced from rPET bottles, offers a sustainable pathway aligned with circular economy goals. Using the kinetic model, we predict the lifetime of virgin PET and 50% rPET blend geotextiles under landfill liner conditions and evaluate their environmental and economic implications (see Materials and Methods).

Lifetime modeling confirmed that both virgin PET and rPET blend geotextile liners retain their structural integrity for more than 50 years under a tensile load *P* = 300 kN/m at pH ≤ 9 (Fig. 6B). As shown in Fig. 6 (C and D), at pH 7 and 9, both environmental and economic performances favor the rPET blend geotextile. The virgin PET configuration exhibits a 100-year global warming potential (GWP-100) of 9.0 ± 0.5 kg CO_2_e/m^2^ and has a cost of about 3.7 ± 0.2 $/m^2^, whereas the rPET blend geotextile achieves 6.4 ± 0.3 kg CO_2_e/m^2^ (30% reduction) and 3.6 ± 0.5 $/m^2^. This indicates a similar cost performance and a substantially reduced carbon footprint. At pH 11, both PET and rPET blend liners experience accelerated hydrolytic degradation, but the impact is far more severe for the rPET geotextile. The service life of rPET blend liner decreases to roughly 7 years, while virgin PET liner retains functionality for about 20 years under the same alkaline conditions, both well below the intended 50-year service life. When these shortened lifetimes are considered, the environmental and economic advantages of using the rPET blend liner disappear. The GWP-100 for the rPET blend liner increases from 6.4 to ∼51 kg CO_2_e/m^2^, accounting for eight replacements over the 50-year period, while its cumulative cost rises from 3.6 $/m^2^ to about 29 $/m^2^. In comparison, the virgin PET liner, requiring replacement three times over the same period, reaches a GWP of ∼27 kg CO_2_e/m^2^ and a total cost near $/m^2^. Therefore, beyond pH 9, the earlier failure of the recycled material negates its initial environmental and cost advantages, as replacement frequency would offset the lower impacts.

Overall, the results indicate that rPET blends are environmentally superior in neutral to mildly alkaline environments. However, under highly alkaline conditions (pH > 9) typical of some landfill leachates, virgin PET remains the more sustainable choice from both an environmental and an economic perspective over an intended 50-year design life. Elevated leachate pH values occur in aged or stabilized landfills, especially those codisposing municipal solid waste with incinerator ash or cementitious residues, where calcium hydroxide dissolution and ammonia formation drive alkalinity increases. Reported leachate pH values for these systems range from 9.2 to 11.5 in US landfills, including sites in Florida, California, and the Midwest that process ash monofills or construction demolition debris (*51*, *52*). In these environments, virgin PET remains the most sustainable and reliable choice for landfill liner applications under such strongly alkaline leachate conditions over a 50-year period.

## DISCUSSION

In this work, we developed an in situ high-throughput photoelasticity platform that enables time-resolved ESC characterization under concurrent mechanical loading and aggressive chemical exposure, overcoming a key limitation of conventional test frames. The setup characterizes multiple specimens simultaneously while preserving independent mechanical responses, reducing testing time by ∼60% and directly capturing crack growth behavior together with evolving stress fields. Applied to alkaline hydrolytic conditions in rPET, we uncover the crack tip stress field evolution, extract crack growth kinetics, and identify ductile-to-brittle transitions that are difficult to access using single-specimen or accelerated aging approaches. These insights reveal that rPET is more vulnerable to environmentally driven mechanical property deterioration compared to virgin PET, exhibiting ∼50% lower ESC resistance, which may reflect recycling-induced microstructural damage. On the basis of the high-throughput dataset, we developed a lifetime prediction framework to support circular economy decisions by identifying deployment conditions under which recycled materials retain durability, safety, and long-term performance.

This work establishes a new experimental paradigm for characterizing materials in complex environments and generating foundational mechanics knowledge. It also illustrates how such data can directly inform sustainability decisions. The platform’s generality opens new pathways for similar assessments across a wide spectrum of emerging sustainable materials, ultimately helping determine when circular materials truly offer net benefits.

## MATERIALS AND METHODS

### Materials and sample preparation

Virgin PET sheet (PET sheet clear, DidiLom, China), a grade commonly used for beverage bottles, with dimensions of 12 by 12 by 0.02 inches (304.8 mm by 304.8 mm by 0.508 mm) was purchased. rPET blend sheet (50% rPET sheet, Wallis Plastic, China) with dimensions of 445 mm by 375 mm by 0.51 mm was also purchased. Solid sodium hydroxide (Sigma-Aldrich, S5881), used to prepare the ESC testing solutions, was purchased from Sigma-Aldrich. All materials were used without any additional pretreatment.

To avoid blunting of the crack tip due to plasticity, the precrack was introduced by a cryogenic cutting method. The samples and the snipper were cooled at −80°C for 15 min. Immediately after removal from the freezer, a crack was introduced at the specimen edge by the snipper. Once the specimen returned to room temperature, the edges were trimmed to obtain the required initial crack length *a*_0_. Microscopic inspection (Exicor MicroImager, Hinds Instruments, OR, USA) confirmed that the crack tip produced by the cryogenic cutting method was sufficiently sharp (fig. S20).

### Fabrication and assembly of high-throughput testing framework

The high-throughput testing framework consists of stainless steel strips and 3D-printed polyethylene terephthalate glycol (PETG) snap joints, assembled with stainless screws and nuts. When tested in pH 13 NaOH solution, the PETG latch structure can maintain the structural and functional integrity for at least 140 hours. The assembly process is straightforward and involves four steps: First, the metal strips are placed in an auxiliary positioning mold, and each specimen is glued between a pair of strips; after fixation, the frame is removed from the mold; the snap joints are then installed onto the frame using screws and nuts to form the latch structure; last, the positions of snap joints are adjusted to ensure latch alignment. Assembling a three-layer high-throughput testing framework requires only about 17 min (movie S3).

### High-throughput tests

All high-throughput experiments were performed under load control using a universal testing machine (Instron E3000 ElectroPuls Dynamic Test System, Instron, MA, USA) equipped with a 1000 N load cell. For monotonic loading, the force was applied at a rate of 2 N s^−1^, and fracture was detected when the load dropped below 10 N, after which the machine held for 3 s before reloading. For long-term ESC tests, the force was increased gradually to the prescribed constant force *F* at a rate of 2 N s^−1^ and then held. When a specimen fractured, the closure of the corresponding latches caused an instantaneous increase in the measured displacement, triggering a 3-s hold followed by reloading to *F*. This sequence continued until all specimens fractured or the total test duration reached 48 hours.

The environmental conditions for ESC testing were controlled by NaOH solutions. Solutions of different concentrations were prepared, and the pH values were measured using pH test strips (Macherey-Nagel, 92110). After the solution was added to the environmental chamber, the chamber was sealed with plastic wrap and a lid to prevent interaction with the atmosphere. At the end of each ESC test, the pH of the solution in the chamber was rechecked using test strips to ensure the validity and stability of the environmental conditions.

In the high-throughput tests, the energy release rate *G* of the single-edge-cracked tensile specimen was calculated by (*53*)

$$G=\left(\pi a\cdot {\sigma_{\text{appl}}}^{2}\cdot \Phi^{2}\right)/E$$ (2)

where *a* is the crack length, σ_appl_ is the applied stress, and Φ is a geometry factor. Consider small-scale yielding condition (*54*), Φ can be expressed as Φ = 1.122 to 0.231(*a*/*w*) + 10.55(*a*/*w*)^2^ – 21.71(*a*/*w*)^3^ + 30.382(*a*/*w*)^4^, where *w* is the initial width of the specimen.

### Kinetic model of hydrolytic cracking

During the slow crack propagation stage of PET hydrolytic cracking, crack growth is governed by the coupling of chemical corrosion and mechanical stress. While phenomenological models are often used to fit experimental crack growth data (*47*, *55*), we extend the Eyring kinetic model to capture the underlying mechanochemical mechanism governing hydrolytic cracking (*48*, *49*). The chemical reacting rate ν is governed by

$$\nu =\nu_{0}\text{exp}\left(-U/\mathrm{kT}\right)$$ (3)

where ν_0_ is the reference reaction rate, *U* represents the reaction energy barrier, *k* is the Boltzmann constant, and *T* is the temperature. Considering that *U* can be reduced by the local stress σ_local_ according to

$$U=U_{0}-{\xi \sigma }_{\text{local}}$$ (4)

where *U*_0_ is the activation energy under zero stress, and ξ is the activation volume. To simplify the model, we assume small-scale yielding, such that within the linear elastic fracture mechanics framework, σ_local_ can be related to the applied energy release rate *G* by (*56*)

$$\sigma_{\text{local}}=\beta \sqrt{G}$$ (5)

where β is a parameter that depends on the material properties and specimen geometry. Therefore, the crack velocity d*a*/d*t* during slow crack propagation, governed by the coupled effects of *G* and chemical reaction kinetics, can be expressed as

$$da/dt=\nu \text{exp}\left(\xi \beta \sqrt{G}/\mathrm{kT}\right)$$ (6)

Here, ν = ν_0_ exp(−*U*_0_/*kT*) refers to the intrinsic chemical reacting rate. Applying the natural logarithm to both sides gives Eq. 1, where α = ξβ/*kT*.

### Lifetime prediction model

Using the kinetic model developed for hydrolytic cracking, we predicted the service lifetime of virgin PET and rPET blend geotextiles under an alkaline environment. We consider a thin, nonwoven, nonporous PET geotextile film of thickness *h* = 1.5 mm, corresponding to a mass of ∼2.7 kg m^−2^ (density of 1.38 g cm^−3^). An initial manufacturing defect of size *a*_0_ = 10^−6^ m is assumed. When the geotextile is subjected to long-term tensile loading in contact with landfill leachate, the defect undergoes hydrolytic crack growth over time. Under an applied tensile load per unit width *P*, the geotextile film sustains an applied stress of *P*/*h*. The energy release rate *G* can be approximated as

$$G=\left(\pi {\mathrm{aP}}^{2}\right)/\left(2{\mathrm{Eh}}^{2}\right)$$ (7)

where the elastic modulus *E* is taken as the experimentally measured value of PET and rPET after 5 days of hydrolytic degradation. The hydrolytic crack growth behavior of the geotextile film is then modeled as

$$da/dt=\left\{\begin{array}{cc} 0 & G<G_{\text{ESC}}\\ \nu \text{exp}\left(\alpha \sqrt{G}\right) & G\geq G_{\text{ESC}} \end{array}\right.$$ (8)

The time to failure *t*_f_ of the geotextile film under a given applied tensile load *P* can then be obtained by integrating the crack growth rate expression as

$$t_{f}=\int_{a_{0}}^{a_{c}}{\left[\nu \text{exp}\left(\alpha \sqrt{G}\right)\right]}^{-1}da$$ (9)

when *G* ≥ *G*_ESC_. Here, *a*_c_ is the critical crack length at which the geotextile loses its structural and functional integrity, set to 10^−2^ m in this case study. To avoid predicting an unphysical infinite lifetime under subcritical loading when *G* < *G*_ESC_, we assigned a minimal constant crack velocity of 10^−14^ m s^−1^, representing the quasi-arrested crack propagation regime. The hydrolytic crack growth of virgin PET and rPET blend geotextile under various pH conditions is shown in fig. S19. Once the crack grows sufficiently for *G* to reach *G*_ESC_, the geotextile enters the slow crack propagation stage, during which the crack velocity increases markedly. The time to failure *t*_f_ is calculated by explicit integration from *a*_0_ to *a*_c_. The predicted *t*_f_ for PET (solid lines) and rPET blend (dashed lines) under different pH conditions is shown in Fig. 6B.

The environmental performance of the geotextile liners was quantified using global warming potentials over a 100-year time horizon (GWP-100), following the 6th Intergovernmental Panel on Climate Change Assessment Report (IPCC AR6) methodology (*57*). Inventory data for PET and rPET production and for film extrusion were obtained from ecoinvent v3.12 (*58*). Market prices were based on 2025 US virgin and recycled polymer resin averages, and industrial-scale film manufacturing cost was also considered. Reported cost data and sources can be found in the Supplementary Materials. The environmental and cost indicators were calculated per square meter of liner over a 50-year service life (functional unit). Calculations were performed for different pH scenarios (*7*, *9*, *11*). The geotextiles were assumed to be buried and protected from ultraviolet. According to lifetime predictions, the service life of the virgin and rPET blend geotextiles was considered as follows: >100 and 80 years at pH 7, 100 and 50 years at pH 9, and 20 and 7 years at pH 11, respectively.

## Supplementary Materials

### The PDF file includes:

Figs. S1 to S20 Tables S1 to S5 Legends for movies S1 to S3 References
