# Supplementary material for: From cracks to informed circularity: Mechanics-guided decisions via high-throughput in situ failure analysis of recycled plastics
Source: Sci Adv. 2026 Jul 23;12(30):eaeh0456. doi: 10.1126/sciadv.aeh0456 (PMC13394384; doi:10.1126/sciadv.aeh0456)
Supplement: Supplementary file 1 — Figs. S1 to S20 Tables S1 to S5 Legends for movies S1 to S3 References [file sciadv.aeh0456_sm.pdf]

Science Advances
AAAS

# Supplementary Materials for

## From cracks to informed circularity: Mechanics-guided decisions via high-throughput in situ failure analysis of recycled plastics

Danqi Sun *et al.*

Corresponding author: Christos E. Athanasiou, athanasiou@gatech.edu

*Sci. Adv.* **12**, eaeh0456 (2026)
DOI: 10.1126/sciadv.aeh0456

**The PDF file includes:**

Figs. S1 to S20
Tables S1 to S5
Legends for movies S1 to S3
References

**Other Supplementary Material for this manuscript includes the following:**

Movies S1 to S3

## Supplementary Text

Fourier transform infrared spectroscopy (FTIR)

We conducted Fourier transform infrared spectroscopy (FTIR) to measure the chemical structures of virgin PET and rPET blend. Both materials were cut into small specimens of 0.6 × 0.6 mm to ensure full contact with the diamond attenuated total reflectance crystal. FTIR spectra were collected by a Nicolet 6700 spectrometer (Thermo Scientific, Madison, WI, USA). Each spectrum was measured over the range of 500-4000 $cm^{-1}$ with a resolution of 4 $cm^{-1}$, with 32 scans averaged to enhance the signal-to-noise ratio. Background spectra were recorded before each measurement to eliminate atmospheric contributions and ensure spectral accuracy.

As shown in Fig. S2, the FTIR spectra of both virgin PET and rPET exhibit the characteristic vibrational signatures of polyethylene terephthalate. The strong absorption band at 1712 $cm^{-1}$ corresponds to the C=O stretching vibration of the ester carbonyl group. The peaks at 1235 $cm^{-1}$ and 1080 $cm^{-1}$ arise from the C-O and C-O-C stretching in the ester linkage, respectively. The band at 1014-1016 $cm^{-1}$ is assigned to the O-$CH_2$ bending vibration of aromatic units in the terephthalate ring. The characteristic peaks at 868-873 $cm^{-1}$ and 722 $cm^{-1}$ are attributed to the benzene ring (59). Overall, the FTIR spectra of rPET and virgin PET show comparable chemical structures, indicating no major changes to the primary ester and aromatic groups after recycling. The blue curves in Fig. S2 show the FTIR spectra of rPET and virgin PET after five days of hydrolytic erosion under pH 13 condition. Compared with the untreated samples, both rPET and virgin PET exhibit increased absorption intensities at ~1235 $cm^{-1}$ and ~1080 $cm^{-1}$. It indicates that the cleavage of ester linkages generates carboxylate and hydroxyl end groups after hydrolysis, enhancing the infrared activity of C-O and C-O-C stretching vibrations.

Differential scanning calorimetry (DSC)

To evaluate the crystallinity of virgin PET and rPET, we conducted differential scanning calorimetry (DSC) measurements using a Discovery X3 DSC (TA Instruments, New Castle, DE, USA). Both materials were cut into small pieces (~8 mg) and sealed in aluminum pans. Each sample underwent a heat-cool cycle under a nitrogen atmosphere. The samples were first heated from 20 °C to 300 °C at 10 °C $min^{-1}$, then cooled to 0 °C at the same rate. The DSC scan results of rPET and virgin PET are shown in Fig. S3. rPET exhibits an indistinct glass transition $T_g$ and broader melting $T_m$ and crystallization $T_c$ peaks compared with virgin PET. These broadened thermal transitions indicate a more heterogeneous polymer chain structure, reflecting a wider molecular weight distribution and increased structural disorder introduced during the recycling process.

The melting enthalpy $\Delta H_m$ and cold crystallization enthalpy $\Delta H_{cc}$ were obtained from the heating curve by integrating the area between the corresponding peak and the baseline. The crystallinity $\chi_c$ is calculated by

$$\chi_c = \frac{\Delta H_m - \Delta H_{cc}}{\Delta H_{m0}} \quad \text{(S1)}$$

where $\Delta H_{m0}$ = 140 J $g^{-1}$ is the melting enthalpy of 100% crystalline PET (60). The results are shown in Table. S5. The crystallinity $\chi_c$ of rPET and virgin PET are 13.4% and 11.2%, respectively.

Uniaxial tensile test

Uniaxial tensile tests were conducted on a universal testing machine (Instron E3000 ElectroPuls™ Dynamic Test System, Instron, MA, USA) equipped with a 1000 N load cell. Plastic sheets were cut into strips with a width of $w = 6$ mm, and sandpaper was bonded to both ends to increase gripping friction. The contour length of the specimen was $l = 45$ mm, and the thickness $h$ was measured by a digital caliper. Tests were performed under displacement control, with the lower grip fixed and the upper grip moving at a rate of 0.5 mm $s^{-1}$. The grip displacement was recorded as x, and the measured force as F. The nominal stress is defined as $\sigma = F/(wh)$, and the engineering strain is defined as $\varepsilon = x/l$. All tests were conducted at room temperature.

The $\sigma$-$\varepsilon$ curves of untreated rPET and virgin PET tested in air are shown in Fig. S4A. Both materials initially show linear elastic deformation. When $\varepsilon > 0.035$, noticeable plastic deformation develops, followed by yielding at $\varepsilon \approx 0.039$. After yielding, the necked region is stretched, with the $\sigma$-$\varepsilon$ responses exhibiting strain softening. Five uniaxial tension tests were conducted for both materials, with the shaded bands representing the variability. Both rPET and virgin PET exhibit scatter around the yield point, while rPET shows slightly greater variability than virgin PET in the post-yield plastic deformation regime.

The elastic modulus $E$ is determined from a linear fit to the $\sigma$-$\varepsilon$ curve for $\varepsilon \leq 0.01$, and the yield stress $\sigma_y$ is defined as the maximum stress in the $\sigma$-$\varepsilon$ curve. $E$ and $\sigma_y$ of virgin PET and rPET are shown in Fig. S4B. rPET exhibits slightly higher values of both properties compared with virgin PET, which may arise from its marginally higher crystallinity.

Fracture toughness measurement

For measuring the toughness of thin ductile sheets, the essential work of fracture (EWF) approach provides a direct and robust methodology (61-63). A series of single-edge-notched specimens with different pre-crack lengths $a_0$ and width $w$ are subjected to uniaxial tension until complete failure (Fig. S5A). The force-displacement ($F$-$x$) curves of rPET and PET are shown in Fig. S5B and Fig. S5C, respectively. The total energy required to fracture a pre-cracked specimen can be separated into two components: the essential work of fracture ($W_e$), associated with creating new surfaces and driving crack propagation, and the plastic work of fracture ($W_p$), which corresponds to energy dissipation in the plastic zone. Accordingly, the total energy $W_f$ can be obtained from the area under the F-x curve and can be expressed by

$$W_f = \int F dx = W_e + W_p \tag{S2}$$

By incorporating the specimen geometry, the fracture energy $w_f$ can be rewritten as

$$w_f = W_f/(Lh) = w_e + \zeta L \cdot w_p \tag{S3}$$

where $L$ is the ligament length, $h$ is the specimen thickness, and $\zeta$ is the shape factor of the plastic zone.

During tensile tests of single-edge-notched specimens, the ligament underwent full yielding prior to crack propagation. The $F$-$x$ curves obtained from specimens with different ligament lengths exhibit geometric similarity, satisfying the prerequisites for applying the EWF method. According to equation (S3), the relationship between $w_f$ and the ligament length $L$ can be obtained, as shown in Fig. S5D and Fig. S5E. The experimental data of $w_f$ as a function of $L$

exhibit a linear relationship, where the slope obtained from linear fitting corresponds to $\zeta w_p$, and the intercept yields $w_e$. Here $w_e$ is a material parameter corresponding to the critical energy required for crack growth and therefore represents the fracture toughness of the material. Using the EWF method, the fracture toughness $w_e$ of rPET is determined to be 8.26 $kJ \cdot m^{-2}$, whereas virgin PET exhibits a much higher value of 22.63 $kJ \cdot m^{-2}$. Although rPET shows slightly higher elastic modulus and yield stress compared with virgin PET, its fracture toughness is substantially lower.

Stress relaxation test

We conducted stress relaxation tests on a universal testing machine (Instron E3000 ElectroPuls™ Dynamic Test System, Instron, MA, USA) equipped with a 1000 N load cell. The dimensions of the tested samples were same as those used for uniaxial tension tests. Each specimen was stretched at a strain rate of 0.001 $s^{-1}$ to $\varepsilon = 0.02$, at which the corresponding stress was recorded as $\sigma_{max}$. The upper grip displacement was then held constant for 15 minutes while the stress relaxation response was recorded. To clearly illustrate how hydrolysis influences the viscoelastic behavior, the stress was normalized to obtain the relationship between the normalized stress $\sigma/\sigma_{max}$ and relaxation time $t_r$, as shown in Fig. S10 and Fig. S11.

Hydrolytic erosion

Hydrolytic degradation of PET is a complex, long-term process involving coupled factors involving coupled effects of penetration, diffusion, and stress distribution (33, 43). To simplify the problem and to calibrate material parameters for subsequent modeling, we first studied the degradation of virgin PET and rPET under stress-free hydrolytic erosion. This approach isolates the chemical contribution by eliminating the influence of stress gradients, allowing the intrinsic changes in mechanical properties to be quantified. Virgin PET and rPET sheets (90 mm in length and 15 mm in width) were fully immersed in deionized water and NaOH solutions of various pH values. Water molecules and hydroxide ions gradually penetrate the polymer network, disrupting physical bonds and inducing base-catalyzed hydrolysis of ester linkages, which leads to material embrittlement. After different durations of hydrolytic erosion, the polymer sheets were removed from solution and subjected to uniaxial tension, photoelasticity and stress relaxation tests to quantify the effects of hydrolysis time on the mechanical properties of the material (Fig. S6).

Hydrolytic degradation in the elastic response was revealed by uniaxial tensile tests. Virgin PET exhibits only minor degradation in $E$ (modulus degradation $\phi_E \approx 10\%$) after 5 days of hydrolytic erosion, while $\sigma_y$ remains relatively stable with only ~5% degradation (Fig. S7). In contrast, rPET undergoes substantially more severe elastic degradation. Its modulus decreases progressively during erosion and stabilizes after approximately one day, reaching $\phi_E \approx 20\%$ relative to the initial value. The $\sigma_y$ of rPET also declines with erosion time, showing a reduction of $\phi_{\sigma y} \approx 10\%$ compared to the original state (Fig. S8). The influence of pH on both material is modest across the tested environments.

To determine whether the stress-optical behavior evolves during hydrolytic cracking, we first examined how the stress-birefringence ($\sigma$-$RGB$) relationship changes during hydrolytic erosion. The evolution of the $\sigma$-$RGB$ curves shows that hydrolysis gradually alters the polymer network configuration in both PET and rPET, with the relationship stabilizing after about one day (Fig. S9). In photoelasticity measurement of hydrolytic crack growth, we adopted the $\sigma$-$RGB$

relationship obtained after five days of hydrolytic erosion, ensuring that the stress field mapping reflect the stabilized optical-mechanical response of the material. For virgin PET, hydrolytic erosion primarily relaxes the residual stress introduced during film processing. The *σ-RGB* relationship shifts relative to the reference standard while maintaining an essentially unchanged slope, suggesting that the polymer chains largely preserve the structural integrity (Fig. S9A). In contrast, rPET shows a more pronounced change in the *σ-RGB* response. The reduced slope reflects a loss of network density caused by hydrolysis-induced chain scission, consistent with the greater molecular-level susceptibility of rPET to degradation (Fig. S9B).

Hydrolytic degradation in the viscoelastic response was evaluated by stress relaxation tests. After hydrolysis, virgin PET exhibits a more pronounced stress decay compared with the untreated material. Water penetration and base-catalyzed hydrolysis disrupt physical intermolecular interactions, facilitate chain slippage, leading to the enhancement of viscous response (45). After about three days, the hydrolytic effect on viscous response reaches saturation. A faster equilibrium of stress relaxation response was observed under lower pH conditions, indicating that water diffusion plays a more dominant role than the hydrolysis reaction (Fig. S10). rPET exhibits comparable stress relaxation behavior across all pH conditions, indicating that water diffusion and hydrolysis make similarly significant contributions to chain slippage.

Fractography characterization

After the ESC tests, the fracture surface was characterized using a a VK-X3000 3D laser scanning microscope (Keyence Corporation, Osaka, Japan). Specimens were removed from the chemical environment, rinsed with ethanol, and stored in sealed bags prior to characterization. During characterization, each specimen was placed on the observation stage, and a rapid navigation scan was first performed using a 10× objective lens. The upper surface of the specimen was then manually focused in laser mode, after which the local region surrounding the crack tip was selected. The 2D profile was captured in camera mode, and the corresponding 3D surface profile was captured in laser mode.

Photoelasticity measurement and data processing

The photoelastic module integrated in the in-situ high-throughput testing platform consists of a white light source, two polarizers (PF030-Linear Polarizer by the foot fully laminated, Polarization), and a 100% blackout light shield (Fig. 2B). The light source is mounted outside the environmental chamber and produces a parallel light field through a diffusion film filter. The incident light passes through the first polarizer, which is installed inside the chamber and sealed by glass to prevent chemical corrosion, with the polarization direction oriented 45° relative to the loading direction of the specimen. When passing through the loaded specimen, the polarized light splits into two light beams with different refractive indices, $n_1$ and $n_2$, sharing the same propagation direction but exhibiting different vibration directions aligned with the two principal stress $\sigma_1$ and $\sigma_2$. These two refracted light beams generate interference on the specimen surface and exhibit a birefringence pattern with a reetardation proportional to the birefringence $\Delta n = n_1 - n_2$, which reflects the local stress state (64-66) (Fig. 2E). Finally, the light exits the observation window, passes through the second polarizer and is captured by a camera (Canon EOS R6 Mark II, equipped with an EF 100 mm f/2.8L Macro lens). for subsequent stress analysis. Depending on the focal length, the resolution of the photoelasticity images ranges from 750 to 900 dpi.

Birefringence was quantified through the *RGB* values of the captured photoelasticity images in this work (42, 50, 67). Photoelasticity calibration was first conducted by uniaxial tension to establish a standard *σ-RGB* relationship. To densify the *σ-RGB* space, the calibrated data points were used as control points for a $C^2$-continuous cubic spline interpolant, producing a smoothed and densely sampled *σ-RGB* manifold. To minimize the influence of imaging hardware and illumination conditions on the birefringence, the *σ-RGB* manifold was converted into the CIE 1931 color space, which decouples chromaticity from luminance and provides a perceptually uniform basis for quantitative stress inference. The resulting manifold serves as a (*σ*, CIE_x, CIE_y) codebook for querying stress values from photoelasticity images.

When processing the non-uniform stress field, the raw photoelasticity image was converted pixel-wise into the CIE 1931 color space. The specimen region was then isolated by removing the dark background through thresholding of the luminance channel. Each pixel within the specimen region subsequently queried the CIE codebook via nearest-neighbor lookup in chromaticity to obtain its corresponding stress value. Since photoelastic fringes are intrinsically periodic, identical chromaticities may correspond to multiple stress levels, resulting in a one-to-many stress mapping when color information is used alone. To resolve this ambiguity, each frame was partitioned into a near-crack zone and a far-field region based on prior knowledge, with each zone assigned upper and lower bounds on admissible stress. During stress-field mapping, each pixel queried the (*σ*, CIE_x, CIE_y) codebook under a region-specific constraint that restricts candidate matches to entries within the prescribed stress bounds. This region-conditioned stress lookup effectively eliminates ambiguity introduced by periodically recurring fringe colors, ensuring accurate local stress assignment and maintaining the global continuity of the reconstructed stress field. Finally, median filtering was applied to the reconstructed stress map to suppress residual impulse-like artefacts while preserving steep stress gradients.

Environmental and economic assessment of the case study

A film thickness of 1.5 mm was assumed, corresponding to a mass of approximately 2.07 $kg \cdot m^{-2}$ (density: 1.38 $g \cdot cm^{-3}$). Inventory data for PET and rPET production, as well as for film extrusion, were sourced from ecoinvent v3.12 (58). For granulate production, the environmental impact is 3.7 kg $CO_2$ per kilogram of virgin PET and 1.17 kg $CO_2$ per kilogram of rPET. Assuming identical emissions during extrusion for both materials, each kilogram of processed geotextile was assigned a uniform emission factor of 0.53 kg $CO_2$. Based on this, the GWP-100 of virgin PET and rPET-blend geotextiles are 9.00 and 6.38 kg $CO_2$ per square meter, respectively.

In terms of cost, the raw material price of virgin PET ranges from 1.2 to 1.6 USD $kg^{-1}$ (68), whereas rPET ranges from 0.625 to 1.572 USD $kg^{-1}$ (69). Assuming a manufacturing cost of 0.3-0.6 USD $kg^{-1}$ for both materials, the corresponding costs per square meter are 3.67 USD for virgin PET and 3.58 USD for rPET-blend geotextiles. We considered a service life unit of 50 years for geotextiles used as landfill liners (70). Based on the lifetime predictions, the required amounts for virgin PET and rPET-blend geotextiles were determined accordingly. Based on the calculations above, the environmental and economic impacts of using virgin PET and rPET-blend geotextiles under different environmental conditions were obtained, as shown in Fig. 6C and Fig. 6D.

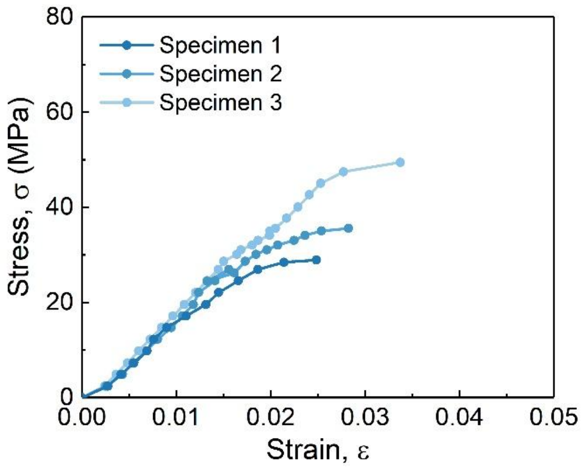


**Fig. S1. Stress-strain curves of individual specimens obtained from the high-throughput single-edge-notched tensile tests.** The initial pre-crack lengths for specimens 1, 2, and 3 are 3.1, 1.9, and 0.8 mm, respectively. Stress $\sigma$ was calculated by dividing the applied force $F$ of the high-throughput testing framework by the cross-sectional area of the specimen. Strain $\varepsilon$ for each specimen was obtained by individually tracking the change in gauge length throughout the loading process.

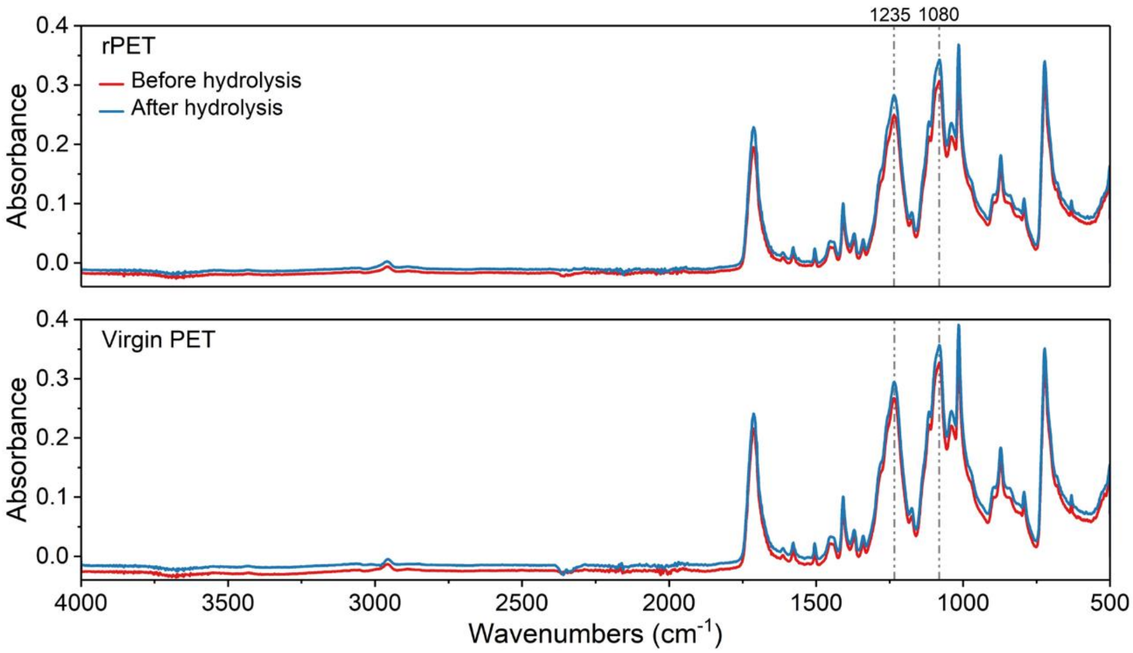


**Fig. S2. Fourier transform infrared (FTIR) spectra of rPET and virgin PET before and after hydrolysis.**

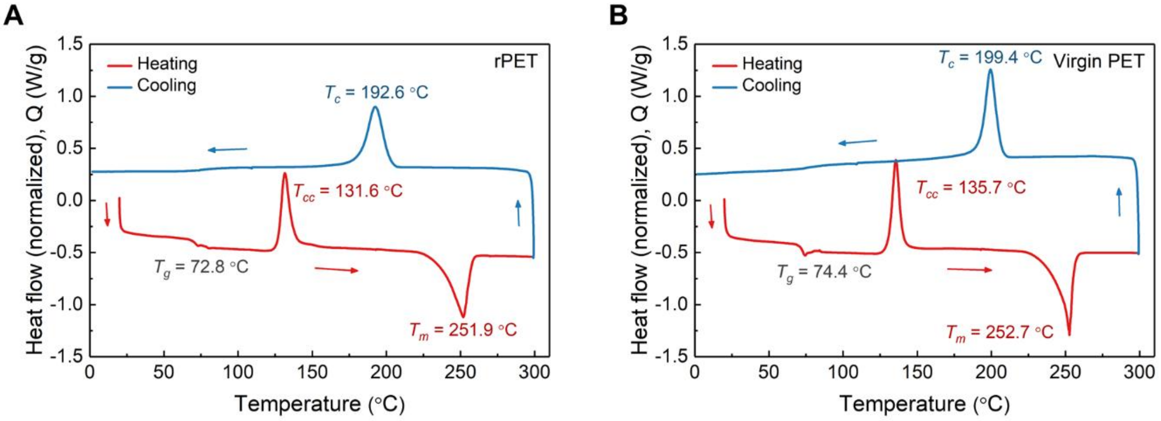


**Fig. S3. Differential scanning calorimetry (DSC) analysis of rPET and virgin PET.** DSC measurement of (**A**) rPET and (**B**) virgin PET.

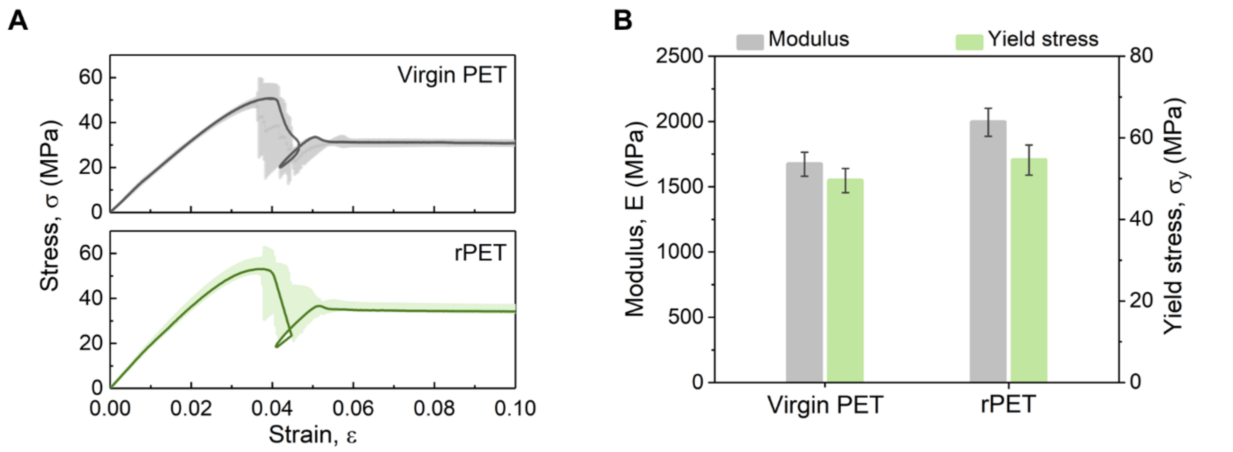


**Fig. S4. Comparison of the mechanical performance of rPET and virgin PET under uniaxial tension.** (**A**) $\sigma$-$\varepsilon$ curves of rPET and virgin PET obtained under uniaxial loading. Both material exhibits yielding and necking at a strain of 0.039, followed by strain softening. The shaded bands indicate the variability among specimens. (**B**) Elastic modulus $E$ and yield stress $\sigma_y$ of virgin PET and rPET. Error bars denote standard deviation (n = 5).

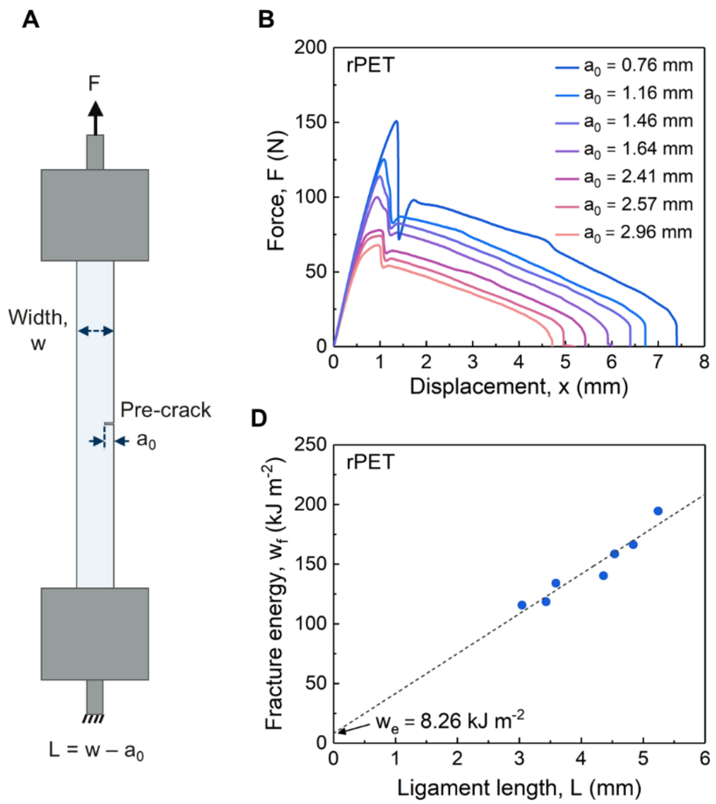


**Fig. S5. Fracture toughness of rPET and virgin PET measured by the essential work of fracture (EWF) method.** (**A**) Schematic of a single-edge-notched specimen with an initial pre-crack length $a_0$ and ligament length $L$. (**B**) Force-displacement curves of rPET specimens with different $a_0$ under uniaxial tension. (**C**) Force-displacement curves of virgin PET specimens with different $a_0$ under uniaxial tension. (**D**) Relationship between fracture energy $w_f$ and ligament length $L$ for rPET. The intercept of the linear fit yields a fracture toughness $w_e$ of 8.26 kJ·m$^{-2}$. (**E**) Relationship between fracture energy $w_f$ and ligament length $L$ for virgin PET. The intercept of the linear fit yields a fracture toughness $w_e$ of 22.63 kJ·m$^{-2}$.

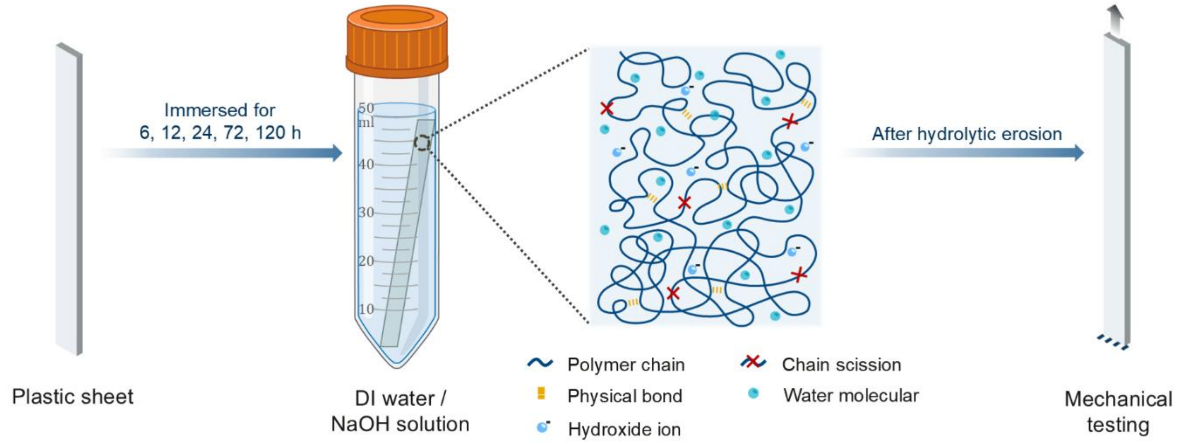


**Fig. S6. Hydrolytic erosion pretreatment of plastic sheets.** Plastic sheets were immersed in DI water or NaOH solution for 6, 12, 24, 72, or 120 hours to induce hydrolytic erosion before mechanical testing.

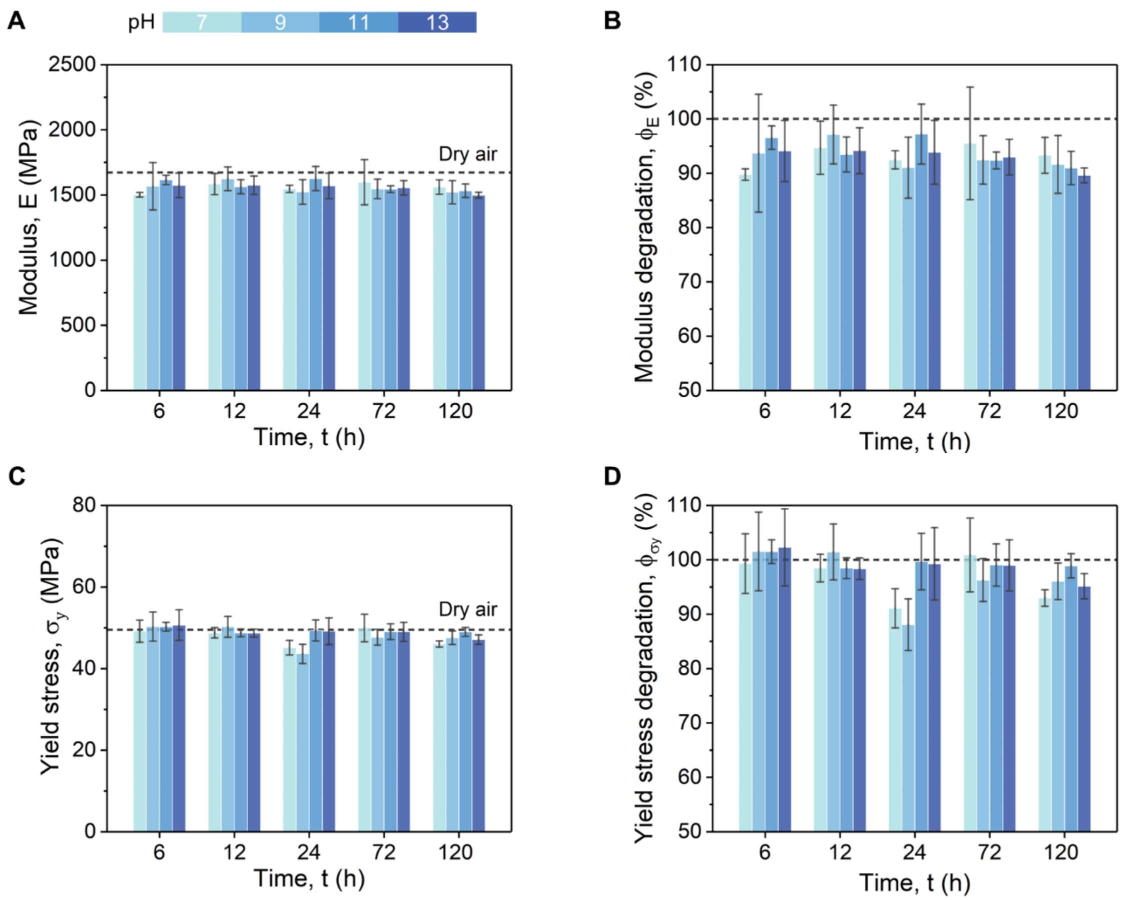


**Fig. S7. Mechanical degradation of virgin PET after hydrolytic erosion for different time under different pH conditions.** (**A**) Elastic modulus $E$. (**B**) Modulus degradation $\phi_E$. (**C**) Yield stress $\sigma_y$. (**D**) Yield stress degradation $\phi_{\sigma y}$. The dashed lines indicate the properties of untreated material. Error bars denote standard deviation (n = 3).

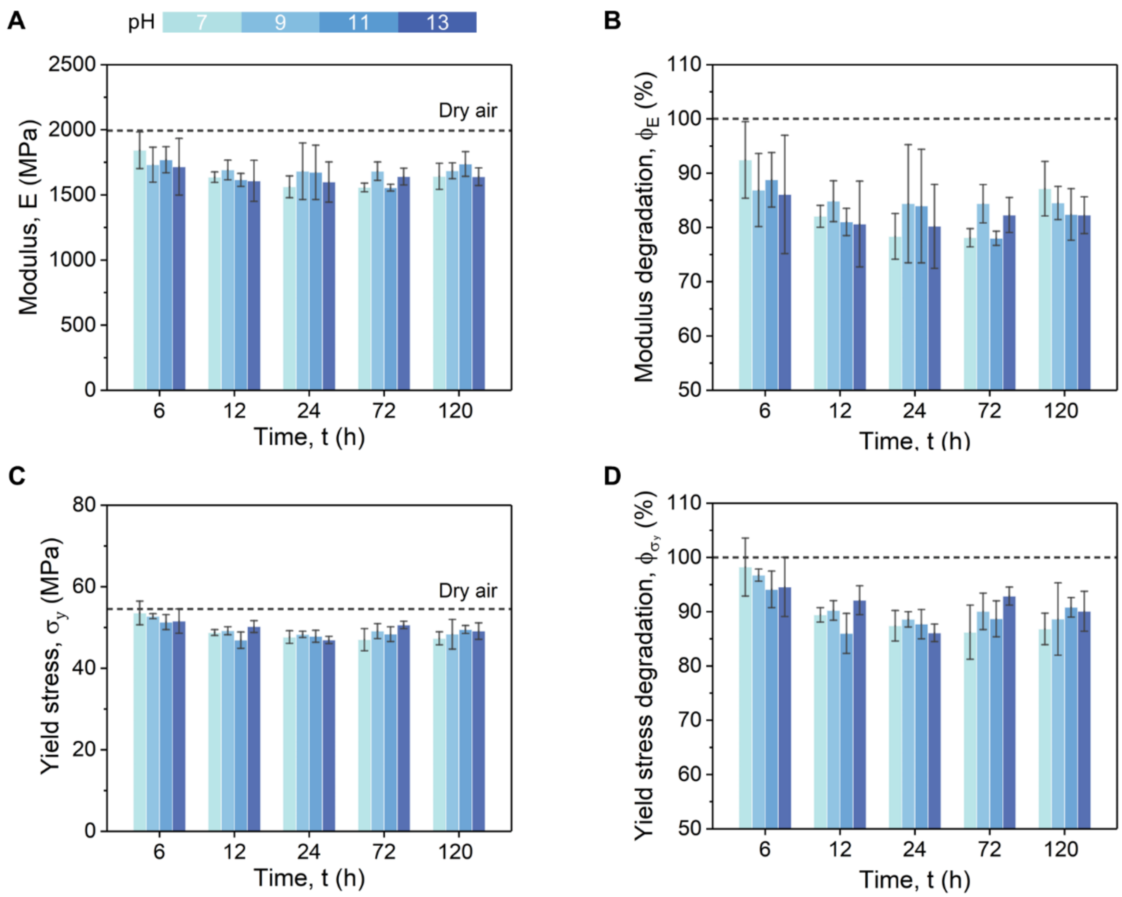


**Fig. S8. Mechanical degradation of rPET after hydrolytic erosion for different time under different pH conditions.** (**A**) Elastic modulus $E$. (**B**) Modulus degradation $\phi_E$. (**C**) Yield stress $\sigma_y$. (**D**) Yield stress degradation $\phi_{\sigma y}$. The dashed lines indicate the properties of untreated material. Error bars denote standard deviation (n = 3).

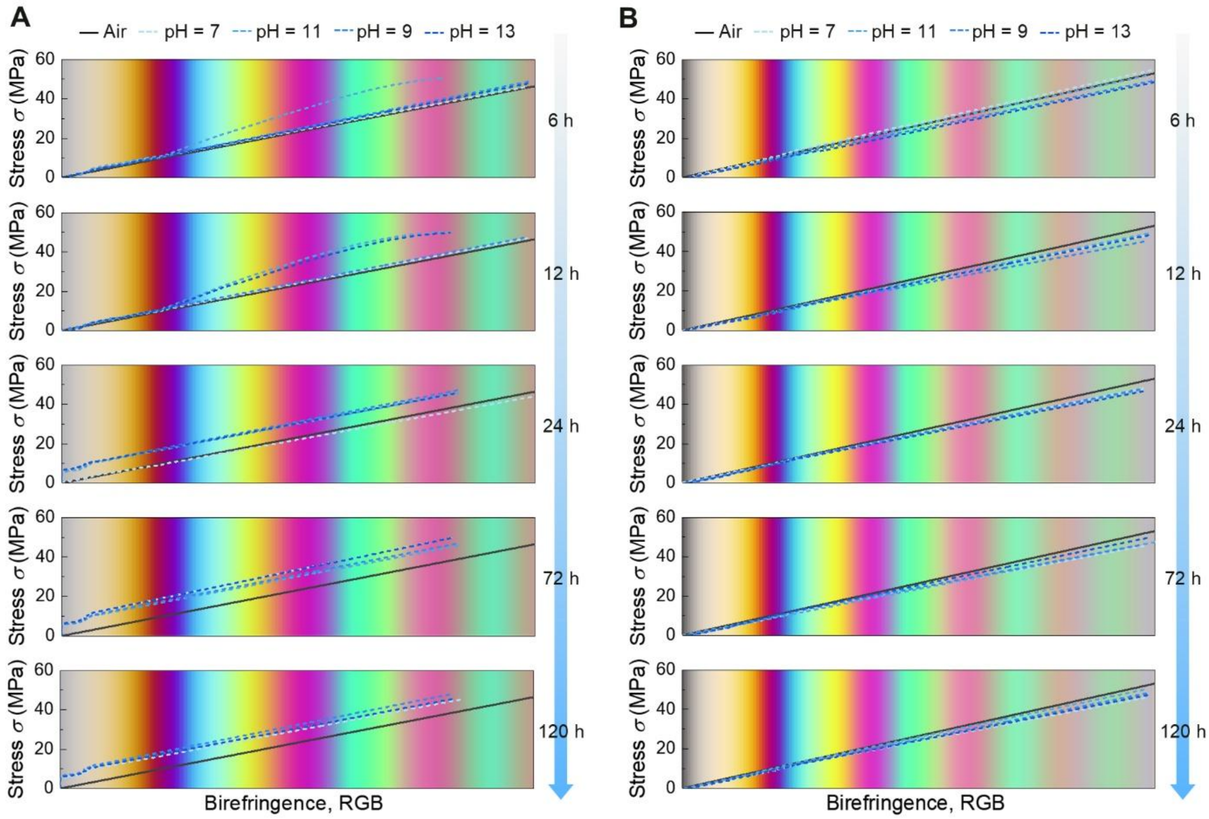


**Fig. S9. Stress-birefringence calibration evolves during hydrolytic erosion.** Stress-birefringence ($\sigma$-$RGB$) relationships for (**A**) virgin PET and (**B**) rPET after hydrolytic erosion in solutions with different pH values for 6, 12, 24, 72, and 120 hours. The air condition is included as a reference. Each curve represents the average obtained from photoelastic measurements of three specimens.

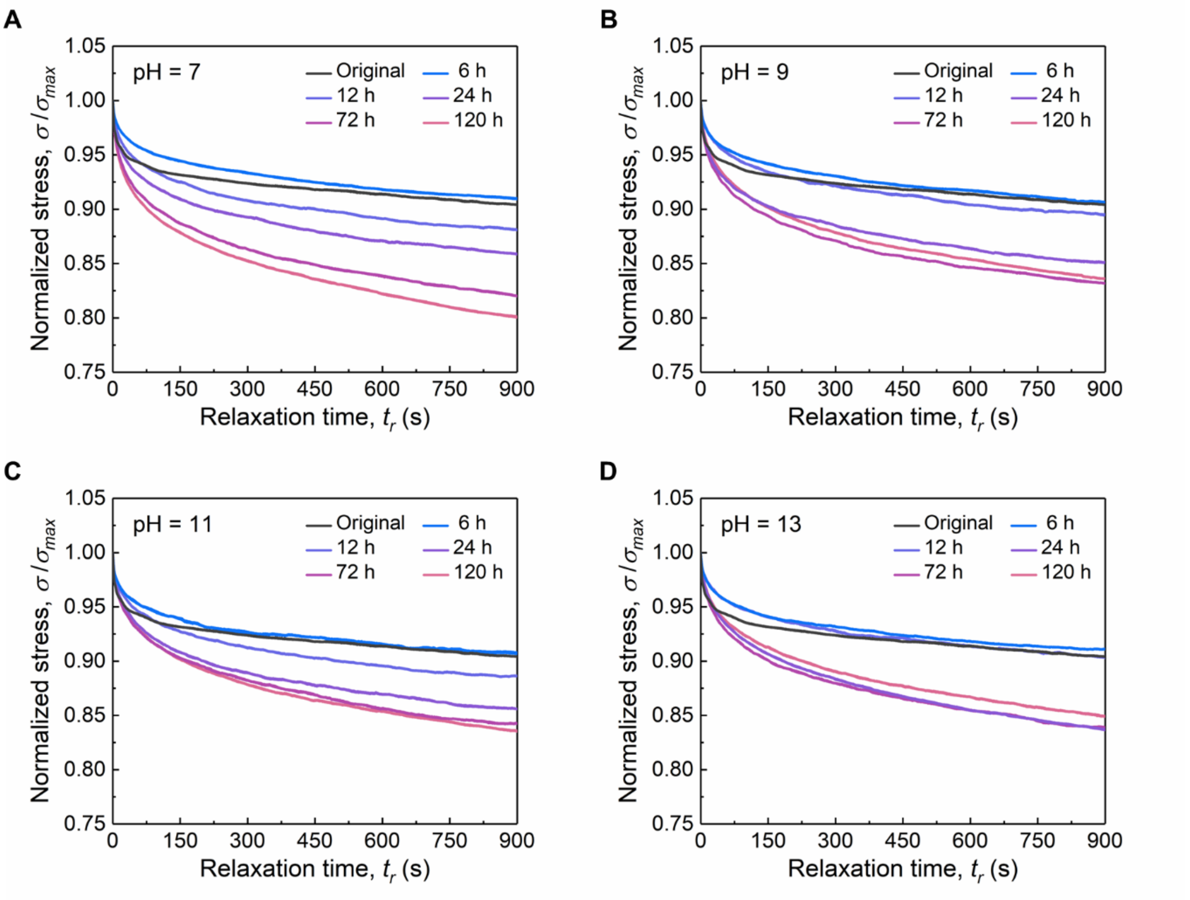


**Fig. S10. Stress relaxation behavior of virgin PET after hydrolytic erosion.** Normalized stress $\sigma/\sigma_{max}$ as a function of relaxation time $t_r$ for virgin PET after hydrolytic erosion for 6, 12, 24, 72, or 120 hours in solutions with (**A**) pH 7, (**B**) pH 9, (**C**) pH 11, and (**D**) pH 13. The original sample is included as a reference.

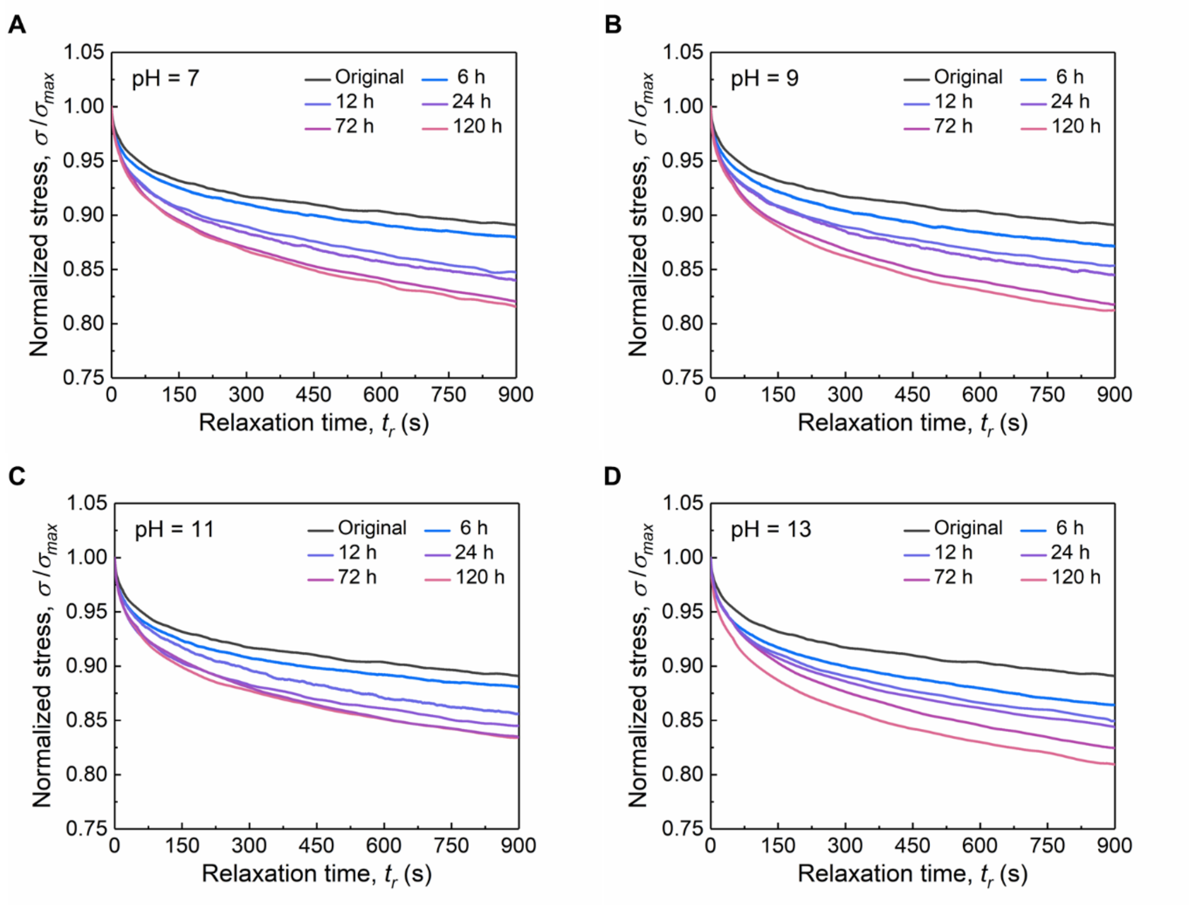


**Fig. S11. Stress relaxation behavior of rPET after hydrolytic erosion.** Normalized stress $\sigma/\sigma_{max}$ as a function of relaxation time $t_r$ for rPET after hydrolytic erosion for 6, 12, 24, 72, or 120 hours in solutions with (**A**) pH 7, (**B**) pH 9, (**C**) pH 11, and (**D**) pH 13. The original sample is included as a reference.

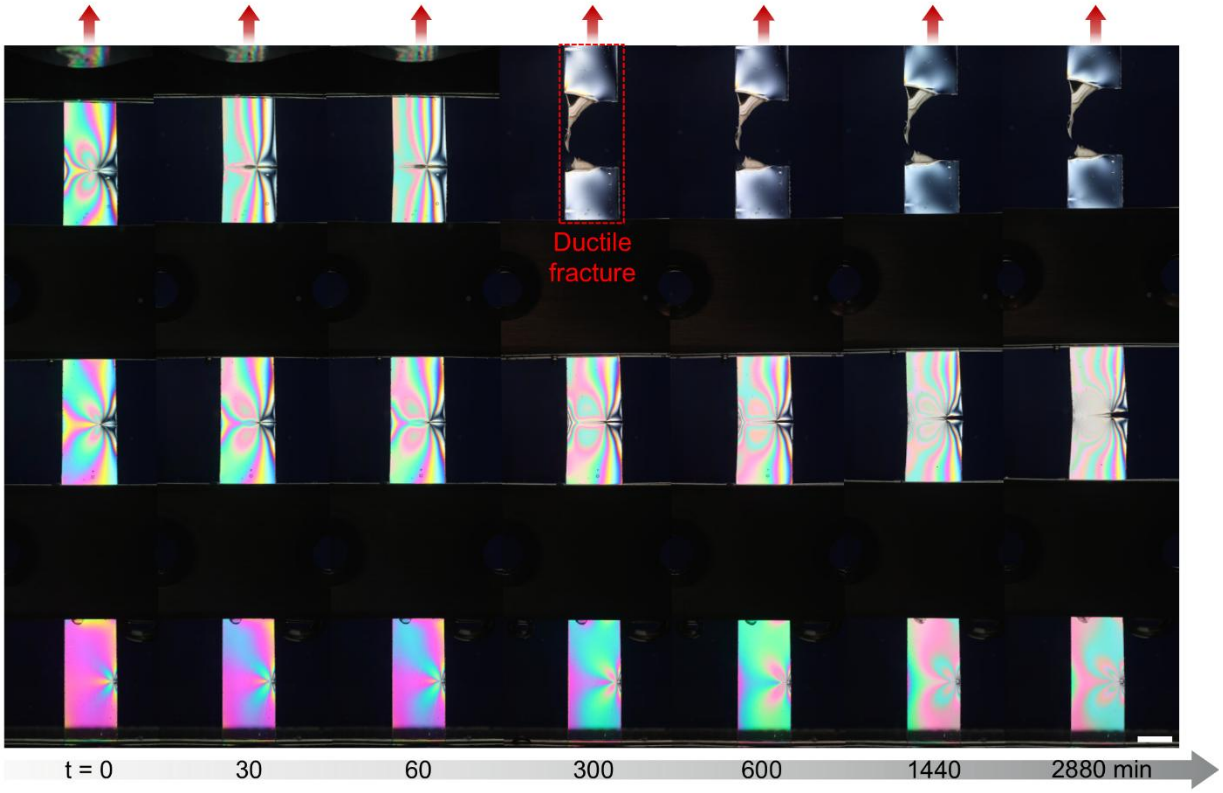


**Fig. S12. High-throughput environmental stress cracking test of rPET under pH 7.** Time-lapse in situ photoelastic images of three rPET specimens tested in pH 7 under an applied force of $F$ = 70 N. All specimens exhibited crack propagation and final failure in a ductile manner. Scale bar: 5 mm.

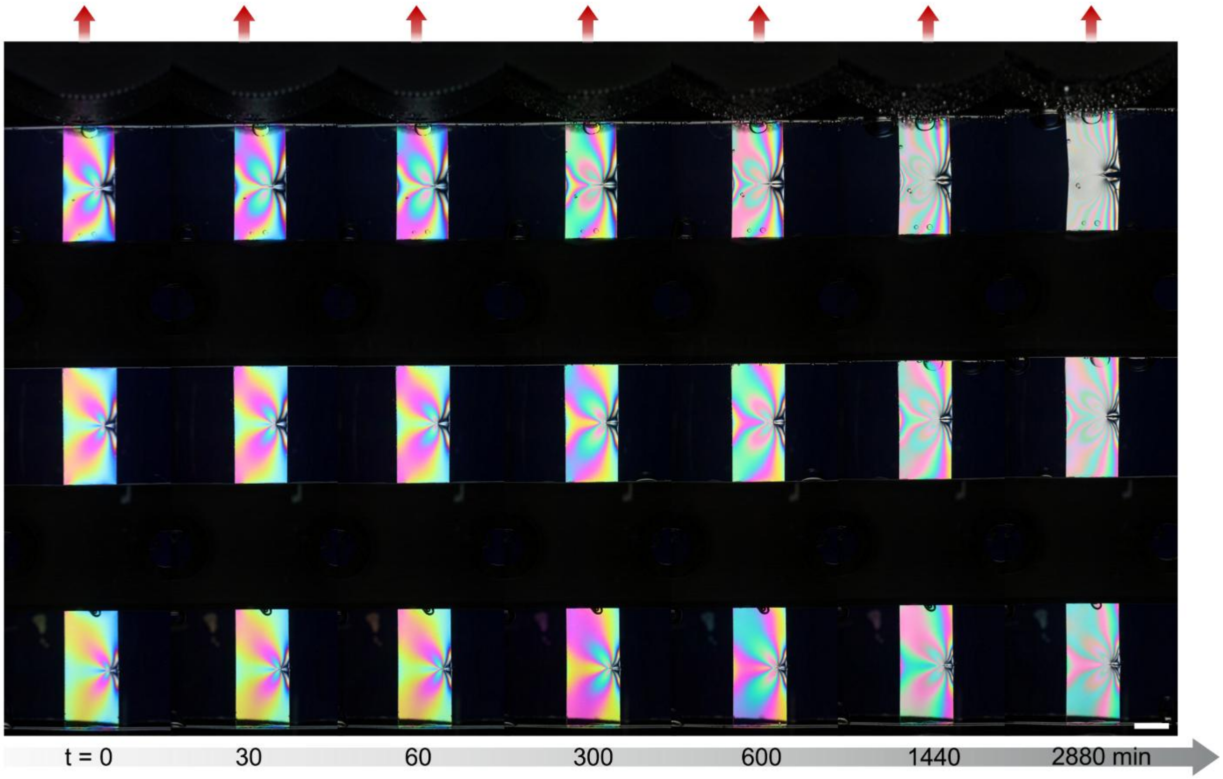


**Fig. S13. High-throughput environmental stress cracking test of rPET under pH 9.** Time-lapse in situ photoelastic images of three rPET specimens tested in pH 9 under an applied force of $F$ = 55 N. All specimens exhibited ductile crack propagation. Scale bar: 5 mm.

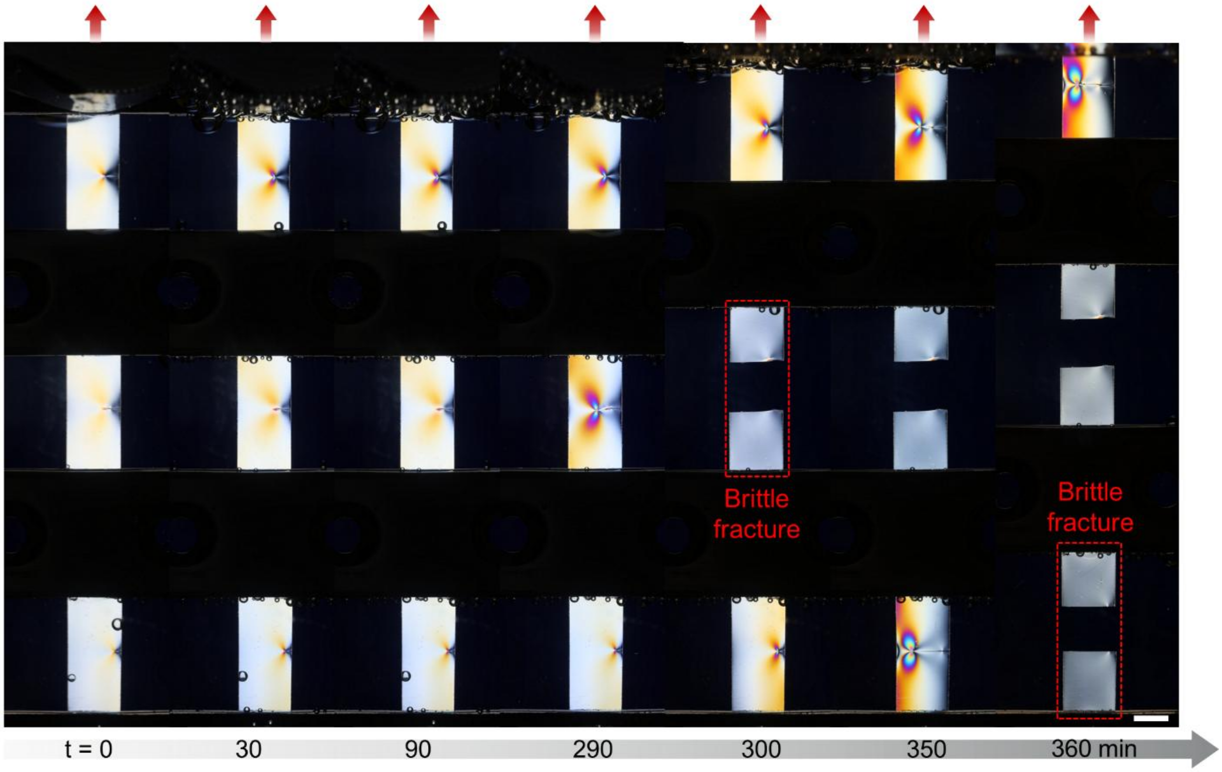


**Fig. S14. High-throughput environmental stress cracking test of rPET under pH 13.** Time-lapse in situ photoelastic images of three rPET specimens tested in pH 13 under an applied force of $F$ = 15 N. All specimens exhibited crack propagation and final failure in a brittle manner. Scale bar: 5 mm.

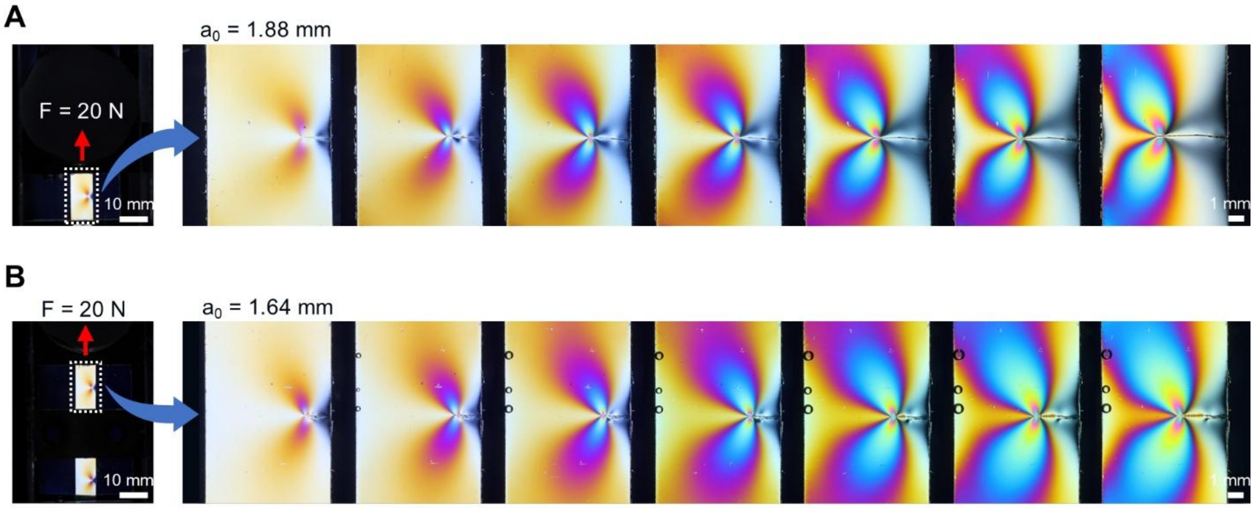


**Fig. S15. Comparison of single-specimen and high-throughput ESC measurements.** Representative photoelastic pattern evolution of rPET during ESC under $F = 20$ N in a pH 11 solution, measured using (**A**) single-specimen setup with initial crack length $a_0 = 1.88$ mm and (**B**) high-throughput setup with initial crack length $a_0 = 1.64$ mm.

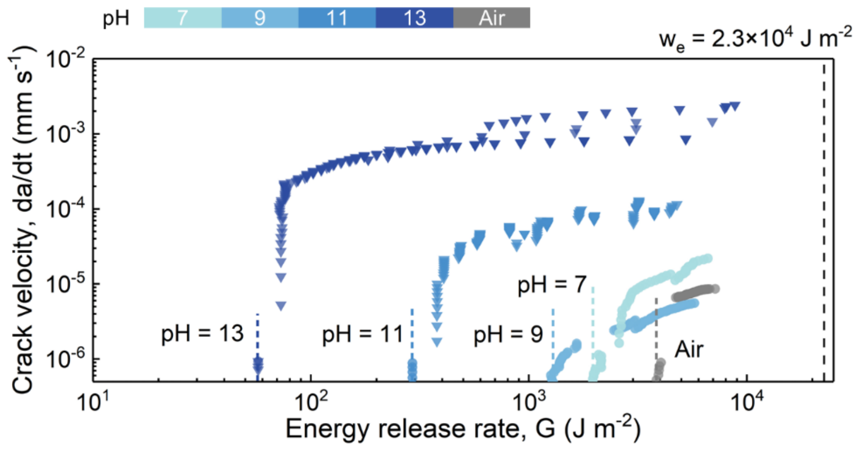


**Fig. S16. Relationship between crack velocity and the applied energy release rate of virgin PET under different environmental conditions.** The fracture toughness $w_e$ of virgin PET under monotonic loading is 22630 $J \cdot m^{-2}$.

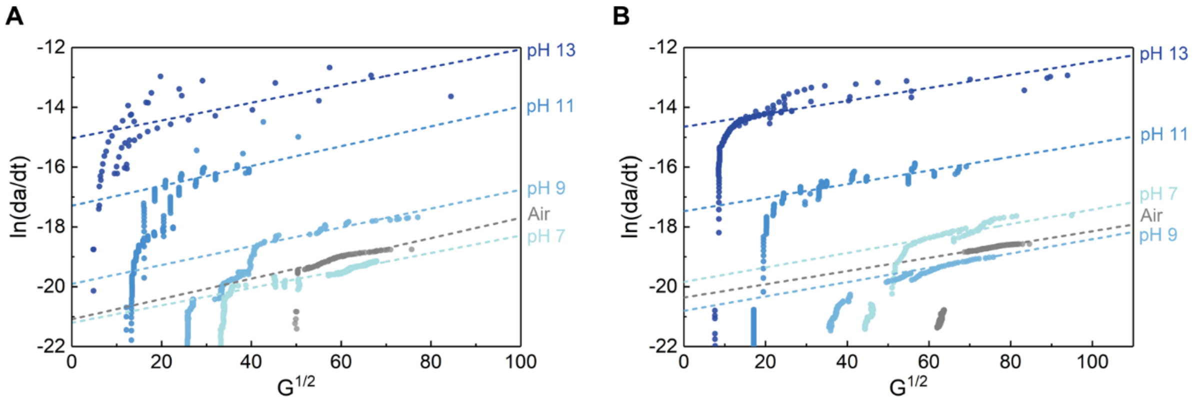


**Fig. S17. Kinetic model fitting of hydrolysis-assisted crack propagation.** Relationship between the natural logarithm of crack velocity ln(d$a$/d$t$) and $G^{1/2}$ under different environmental conditions for (**A**) rPET and (**B**) virgin PET. The dashed lines represent the kinetic model fits in the ESC slow crack propagation regime.

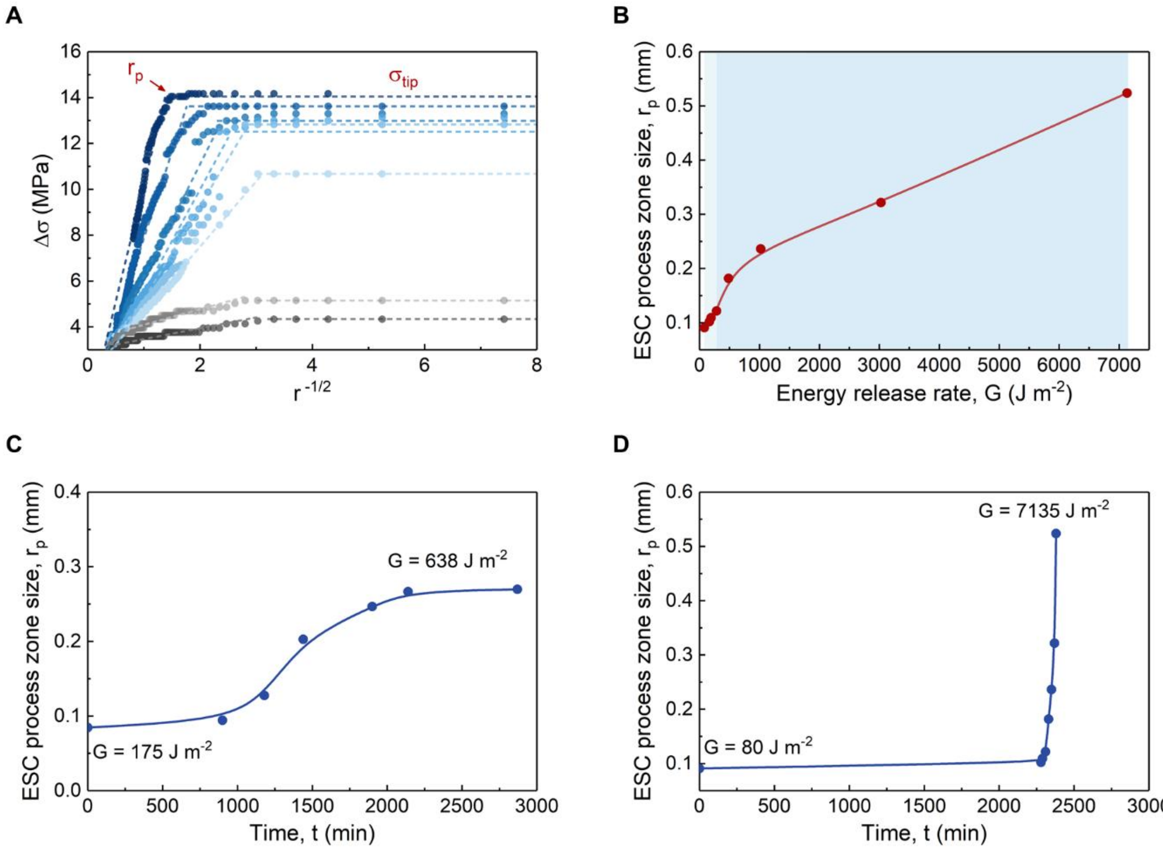


**Fig. S18. Evolution of crack-tip stress during hydrolysis-driven brittle fracture under highly alkaline conditions.** (**A**) Relationship between the stress difference $\Delta\sigma$ and $r^{1/2}$ at $\theta = 60°$ under pH 13. The applied energy release rate $G$ increases from 80 to 7135 J·m$^{-2}$. (**B**) Evolution of the ESC process zone size $r_p$ with $G$ under pH 13. The ESC process zone size increases slowly from 0.09 to 0.12 mm (38.5 hours), then expands rapidly to 0.52 mm within 1.1 hour until complete failure. (**C**) Evolution of $r_p$ with time $t$ during hydrolytic cracking under pH 11. The ESC process zone undergoes slow expansion, rapid expansion, and finally approaches a plateau. (**D**) Evolution of $r_p$ with time $t$ during hydrolytic cracking under pH 13. Under a more strongly alkaline environment, the ESC process zone undergoes slow expansion and rapid expansion, and fracture occurs before reaching equilibrium.

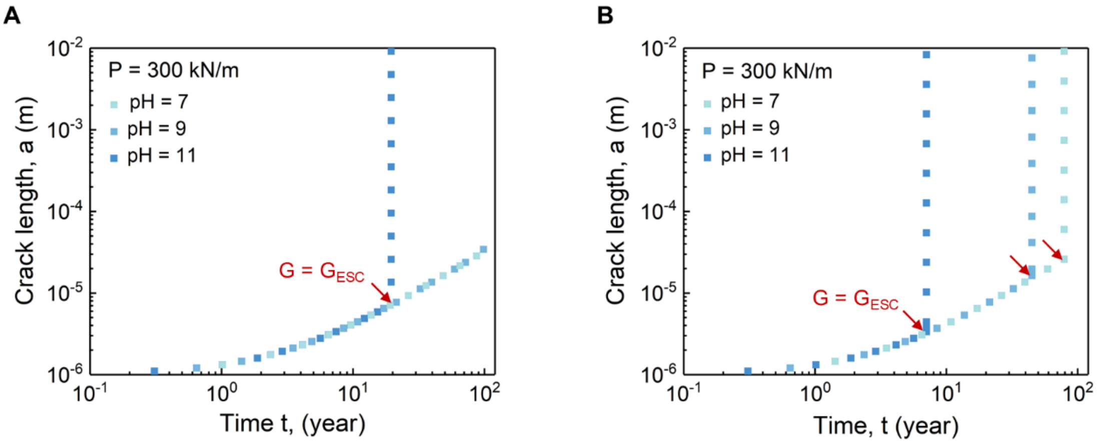


**Fig. S19. Evolution of crack length in geotextiles used as landfill liners.** Assuming a tensile load of $P$ = 300 kN m$^{-1}$ per unit width, $a$ is shown as a function of service time $t$ for (**A**) virgin PET geotextile and (**B**) rPET-blend geotextile under pH 7, 9 and 11. When $G$ reaches $G_{ESC}$, the crack transitions from the quasi-arrested crack propagation into the slow crack propagation stage.

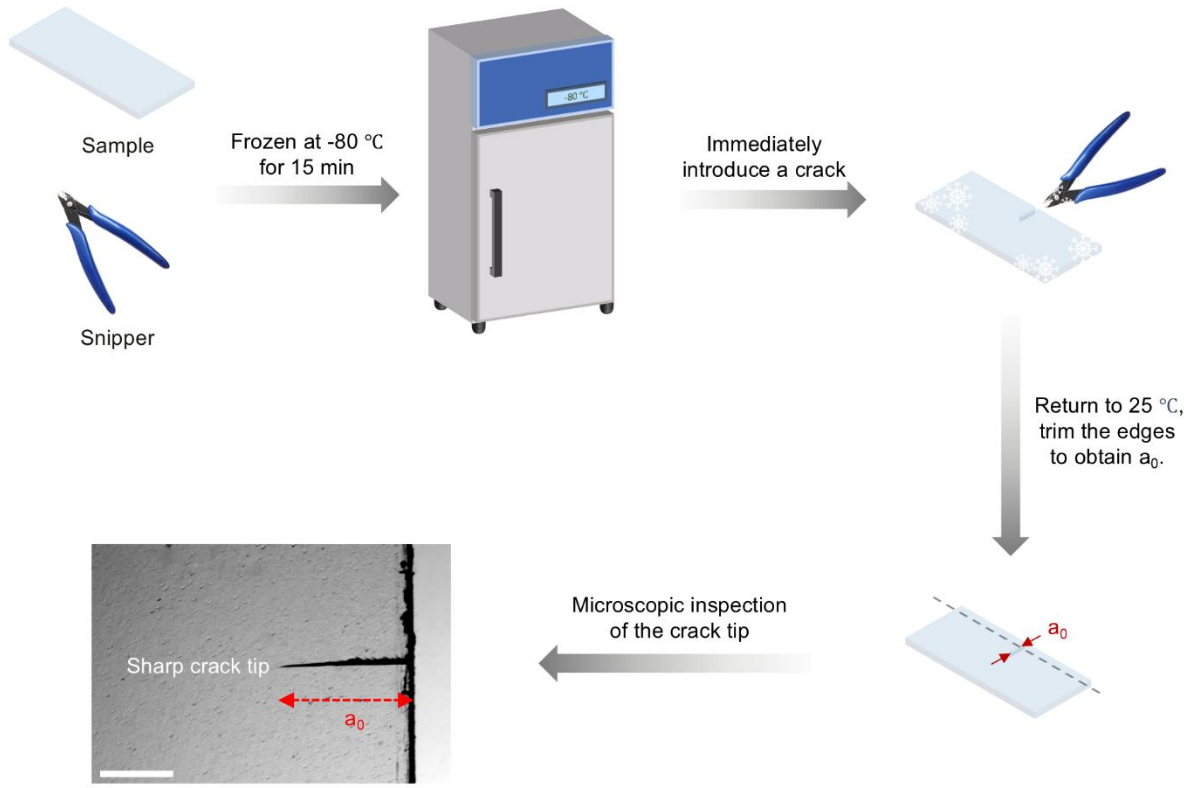


**Fig. S20. Schematic illustration of the procedure for introducing a single-edge pre-crack using the cryo-cutting method.** Microscope image was captured by Exicor MicroImager$^{TM}$. scale bar: 1 mm.

**Table S1.** Comparison of key features of existing high-throughput environmental testing systems

| Material type | Loading condition | Number of samples | Sequential (one specimen per run) / Simultaneous (multiple specimens tested at once) | Environ-mental control module | In-situ measure-ment | Key novelty | Reference |
|---|---|---|---|---|---|---|---|
| Thin metal, plastic, rubber and composite specimens | Tensile | 6 | Simultaneous | No | No | Consolidate test stations. | (71) |
| Not specified | Fatigue | 16 | Simultaneous | Heat-controlled fluid bath | Optical measure-ments | Simultaneous axial fatigue of 16 specimens. | (72) |
| Not specified | Tensile | 50 | Sequential | No | Camera | High-throughput sequential tensile testing. | (73, 74) |
| Not specified | Cyclical tensile loading | 10 | Simultaneous | Atmos-pheric agents | Sensors for measuring crack | Applicable for parallel fatigue testing of multiple specimens in corrosive environments. | (75) |
| 3D printed materials, composites, alloy | Small punch | 100+ | Sequential | No | No | Automated pinpoint clamping and testing mechanism. | (76) |
| 3D printed polymers | Additive manu-facturing | Multiple | Simultaneous | Yes | Environm ental, stress and impedan-ce sensor. | In-situ screening during additive manufactur-ing. | (77) |
| Cell culture | Physiologic waveform of mechanical strain | 6 | Simultaneous | Cell culture substrate | No | Application of physiological-ly relevant strain signals to cell cultures. | (78) |

| | | | | | | | |
|---|---|---|---|---|---|---|---|
| 3D printed composites | Tensile | 4 | Simultaneous | No | No | Load-controlled high-throughput strength testing using redundant load-carrying tape. | (79) |
| 316 L stainless steel | Creep | 18 | Simultaneous | No | No | Parallel room-temperature creep testing via shared connector and varied cross-sections. | (18) |
| 3D printed elastomer | Tensile, fatigue | 1000 | Simultaneous | No | Camera | Enabling fatigue testing of 1000 3D-printed specimens through displacement-controlled loading and specially designed clamping structure. | (80-82) |
| Thin films | Monotonic tensile and fatigue testing | 6-12 | Simultaneous | No | DIC, in-situ SEM | Quantify statistical variations in degradation mechanism at micro- and nanoscale | (83) |
| Alloy thin films | Residual stress | 50 | Simultaneous | Heating stage | Camera | A special substrate curvature-based apparatus. | (84) |

| | | | | | | | |
|---|---|---|---|---|---|---|---|
| Polymer | Depth-sensing indentation (DSI) | 500-1700 | Sequential | No | Atomic force micro-scope | Enables quantitative analysis of modulus, creep, and yield properties in small volumes of polymeric materials. | (19, 26) |
| Polymer film | Impact tests | 100 | Sequential | No | No | Electro-magnetic testing system enables dynamic mechanical testing from near-static to high-speed impact. | (85) |
| Thin-film array | Bulge | 100 | Simultaneous | No | DIC | Simultaneous measurement of elastic modulus and Poisson's ratio of 10×10 thin-film array by bulge method. | (86) |
| Soft polymer | Centrifuga-tion | 1536 | Simultaneous | No | No | A centrifuge-based method for high-throughput testing of fracture stress of soft polymer in 1536-well plate. | (25) |
| Polymer | Tensile, creep, environ-mental stress cracking | 3+ | Simultaneous | Chemical solution, controlled tempera-ture and humid gas | In-situ Photo-elasticity | In-situ stress measurement and multi-specimen environmental testing. | This work |

**Table S2.** Summary of recycled PET specimens tested under various environmental conditions.

| Environmental condition | Specimen index | Initial crack length $a_0$ [mm] | Applied stress $\sigma_{appl}$ [MPa] | Initial energy release rate $G_0$ [kJ m$^{-2}$] |
|---|---|---|---|---|
| Air | R-a-1 | 1.57 | 23.28 | 2.53 |
| | R-a-2 | 1.79 | 20.83 | 2.51 |
| | R-a-3 | 1.13 | 23.28 | 1.53 |
| pH 7 | R-7-1 | 2.90 | 17.16 | 5.99 |
| | R-7-2 | 2.47 | 18.87 | 4.81 |
| | R-7-3 | 2.51 | 17.65 | 4.38 |
| | R-7-4 | 2.26 | 17.16 | 3.27 |
| | R-7-5 | 1.06 | 17.65 | 0.97 |
| | R-7-6 | 0.71 | 18.87 | 0.66 |
| pH 9 | R-9-1 | 1.68 | 15.93 | 1.55 |
| | R-9-2 | 1.89 | 13.48 | 1.38 |
| | R-9-3 | 1.70 | 13.48 | 1.13 |
| | R-9-4 | 1.26 | 15.93 | 0.98 |
| | R-9-5 | 1.21 | 13.48 | 0.66 |
| pH 11 | R-11-1 | 1.98 | 15.93 | 2.04 |
| | R-11-2 | 2.37 | 11.11 | 1.44 |
| | R-11-3 | 1.70 | 8.33 | 0.42 |
| | R-11-4 | 1.26 | 8.33 | 0.26 |
| | R-11-5 | 1.64 | 5.56 | 0.18 |
| | R-11-6 | 1.48 | 5.56 | 0.15 |
| pH 13 | R-13-1 | 2.20 | 3.68 | 0.14 |
| | R-13-2 | 2.64 | 2.94 | 0.14 |
| | R-13-3 | 1.87 | 3.68 | 0.10 |
| | R-13-4 | 2.46 | 2.45 | 0.08 |
| | R-13-5 | 1.28 | 2.94 | 0.04 |
| | R-13-6 | 1.89 | 1.72 | 0.02 |

**Table S3.** Summary of virgin PET specimens tested under various environmental conditions.

| Environmental condition | Specimen index | Initial crack length $a_0$ [mm] | Applied stress $\sigma_{appl}$ [MPa] | Initial energy release rate $G_0$ [kJ m$^{-2}$] |
|---|---|---|---|---|
| Air | V-a-1 | 1.88 | 25.00 | 4.72 |
| | V-a-2 | 2.19 | 19.44 | 3.85 |
| | V-a-3 | 1.00 | 29.17 | 2.39 |
| pH 7 | V-7-1 | 3.39 | 18.33 | 11.64 |
| | V-7-2 | 2.50 | 17.22 | 4.37 |
| | V-7-3 | 2.19 | 18.89 | 3.91 |
| | V-7-4 | 1.94 | 17.22 | 2.54 |
| | V-7-5 | 1.68 | 17.22 | 1.95 |
| | V-7-6 | 1.20 | 17.22 | 1.15 |
| pH 9 | V-9-1 | 2.23 | 16.11 | 3.03 |
| | V-9-2 | 2.24 | 14.44 | 2.46 |
| | V-9-3 | 1.71 | 14.44 | 1.45 |
| | V-9-4 | 1.39 | 16.11 | 1.29 |
| | V-9-5 | 0.99 | 15.56 | 0.73 |
| pH 11 | V-11-1 | 2.56 | 15.28 | 3.69 |
| | V-11-2 | 2.77 | 12.50 | 3.02 |
| | V-11-3 | 2.06 | 15.28 | 2.30 |
| | V-11-4 | 1.92 | 10.56 | 0.95 |
| | V-11-5 | 2.39 | 8.06 | 0.87 |
| | V-11-6 | 2.49 | 5.28 | 0.41 |
| | V-11-7 | 2.12 | 5.28 | 0.29 |
| pH 13 | V-13-1 | 2.71 | 6.94 | 0.90 |
| | V-13-2 | 2.88 | 4.44 | 0.43 |
| | V-13-3 | 2.59 | 5.00 | 0.41 |
| | V-13-4 | 1.59 | 6.94 | 0.30 |
| | V-13-5 | 2.82 | 3.33 | 0.23 |

| | | | |
|---|---|---|---|
| V-13-6 | 1.94 | 4.44 | 0.18 |
| V-13-7 | 0.95 | 5.00 | 0.07 |
| V-13-8 | 0.94 | 4.44 | 0.06 |

**Table S4.** Parameters of the kinetic model under different environmental conditions.

| Environmental condition | Intrinsic chemical reacting rate $v$ [m $s^{-1}$] | | Mechanical effect factor $\alpha$ [$(Nm)^{1/2}$ $s^{-1}$] | |
|---|---|---|---|---|
| | rPET | Virgin PET | rPET | Virgin PET |
| Air | 6.9E-10 | 1.4E-09 | 0.034 | 0.022 |
| pH 7 | 6.1E-10 | 2.4E-09 | 0.029 | 0.024 |
| pH 9 | 2.3E-09 | 9.3E-10 | 0.032 | 0.024 |
| pH 11 | 3.1E-08 | 2.6E-08 | 0.033 | 0.023 |
| pH 13 | 2.9E-07 | 4.3E-07 | 0.030 | 0.022 |

**Table S5.** The crystallinity of rPET and virgin PET measured by DSC.

| Sample | $T_m$ [°C] | $\Delta H_m$ [J g$^{-1}$] | $T_{cc}$ [°C] | $\Delta H_{cc}$ [J g$^{-1}$] | $\chi_c$ [%] |
|---|---|---|---|---|---|
| rPET | 251.9 | 49.5 | 131.6 | 30.8 | 13.4 |
| PET | 252.7 | 49.6 | 135.7 | 33.9 | 11.2 |

**Movie S1.** High-throughput fracture testing of three single-edge-notched specimens.

**Movie S2.** High-throughput ESC testing of rPET specimens under pH 11.

**Movie S3.** Assembly of the high-throughput testing framework.

## REFERENCES


1. J. Steck, J. Kim, Y. Kutsovsky, Z. Suo, Multiscale stress deconcentration amplifies fatigue resistance of rubber. *Nature* **624**, 303–308 (2023).

2. G. E. Sanoja, X. P. Morelle, J. Comtet, C. J. Yeh, M. Ciccotti, C. Creton, Why is mechanical fatigue different from toughness in elastomers? The role of damage by polymer chain scission. *Sci. Adv.* **7**, eabg9410 (2021).

3. S. Lin, X. Liu, J. Liu, H. Yuk, H.-C. Loh, G. A. Parada, C. Settens, J. Song, A. Masic, G. H. McKinley, X. Zhao, Anti-fatigue-fracture hydrogels. *Sci. Adv.* **5**, eaau8528 (2019).

4. Y. Zhou, L. Jin, Hydrolysis-induced large swelling of polyacrylamide hydrogels. *Soft Matter* **16**, 5740–5749 (2020).

5. S. Konica, T. Sain, A thermodynamically consistent chemo-mechanically coupled large deformation model for polymer oxidation. *J. Mech. Phys. Solids* **137**, 103858 (2020).

6. N. M. Ames, V. Srivastava, S. A. Chester, L. Anand, A thermo-mechanically coupled theory for large deformations of amorphous polymers. Part II: Applications. *Int. J. Plast.* **25**, 1495–1539 (2009).

7. H. Chen, Z. Pan, D. Yuan, G. S. Sulley, R. N. Oosterbeek, C. K. Williams, L. Brassart, Shear yielding and crazing in dry and wet amorphous PLA at body temperature. *Polymer* **289**, 126477 (2023).

8. K. V. Vaishakh, N. K. Parambil, V. Srivastava, Hygroscopic damage of fiber–matrix interface in unidirectional composites: A computational approach. *Int. J. Mech. Sci.* **279**, 109460 (2024).

9. X. Q. Wang, G. X. Gu, Environment-process-structure-property linkages in additive manufacturing. *Nat. Rev. Clean Technol.* **1**, 751–752 (2025).

10. A. K. Rodriguez, B. Mansoor, G. Ayoub, X. Colin, A. A. Benzerga, Effect of UV-aging on the mechanical and fracture behavior of low density polyethylene. *Polym. Degrad. Stab.* **180**, 109185 (2020).

11. P. M. Stathatou, C. E. Athanasiou, M. J. Realff, From circularity to spirality: An integrated, systems-level approach to address the plastics problem. *J. Am. Chem. Soc.* **147**, 32299–32308 (2025).

12. K. D. Nixon, Z. O. Schyns, Y. Luo, M. G. Ierapetritou, D. G. Vlachos, L. T. Korley, T. H. Epps III, Analyses of circular solutions for advanced plastics waste recycling. *Nat. Chem. Eng.* **1**, 615–626 (2024).

13. D. Georgiou, D. Sun, X. Liu, C. E. Athanasiou, Suppressing mechanical property variability in recycled plastics via bioinspired design. *Proc. Natl. Acad. Sci. U.S.A.* **122**, e2502613122 (2025).

14. D. Georgiou, D. Okegbu, Z. Yang, T. Wang, M. R. Snowdon, A. Mohanty, N. Gershenfeld, W. Yan, C. E. Athanasiou, Eco-voxels: Building blocks for sustainable, load-bearing structures. *Matter* **8**, 102106 (2025).

15. J. Lu, K. Ravi-Chandar, Inelastic deformation and localization in polycarbonate under tension. *Int. J. Solids Struct.* **36**, 391–425 (1999).

16. A. P. Taylor, G. S. Vankayalapati, M. K. Budzik, K. T. Turner, Hinged rigid beam fracture specimen for characterization of lattice and thin-sheet materials. *Exp. Mech.* **65**, 1375–1386 (2025).

17. G. H. Lim, M. J. Choi, C. H. Lee, S. J. Choi, N. I. Kim, J. W. Wee, Quantitative in-situ evaluation of environmental stress cracking resistance in poly(methyl methacrylate) using automated crack detection. *Polym. Test.* **152**, 108998 (2025).

18. S. B. Inman, K. W. Garber, A. E. Robertson, N. K. Brown, R. Dingreville, B. L. Boyce, Stochastic room temperature creep of 316 L stainless steel. *Int. J. Plast.* **189**, 104326 (2025).

19. J. M. Kranenburg, C. A. Tweedie, K. J. van Vliet, U. S. Schubert, Challenges and progress in high-throughput screening of polymer mechanical properties by indentation. *Adv. Mater.* **21**, 3551–3561 (2009).

20. T. Chen, Z. Pang, S. He, Y. Li, S. Shrestha, J. M. Little, H. Yang, T. C. Chung, J. Sun, H. C. Whitley, I. C. Lee, T. J. Woehl, T. Li, L. Hu, P. Y. Chen, Machine intelligence-accelerated discovery of all-natural plastic substitutes. *Nat. Nanotechnol.* **19**, 782–791 (2024).

21. S. Oh, E. E. Stache, Recent advances in oxidative degradation of plastics. *Chem. Soc. Rev.* **53**, 7309–7327 (2024).

22. M. X. Z. Shi, J. Steck, X. X. Yang, G. G. Zhang, J. Yin, Z. G. Suo, Cracks outrun erosion in degradable polymers. *Extreme Mech. Lett.* **40**, 100978 (2020).

23. A. D. Patel, Z. O. G. Schyns, T. W. Franklin, M. P. Shaver, Defining quality by quantifying degradation in the mechanical recycling of polyethylene. *Nat. Commun.* **15**, 8733 (2024).

24. D. O. Kazmer, S. O. Nzeh, B. Shen, D. C. Elbert, R. Nagarajan, M. Sobkowicz-Kline, T. D. Nguyen, Characterization, processing, and modeling of industrial recycled polyolefins. *Polym. Eng. Sci.* **64**, 4801–4815 (2024).

25. J. E. Griffith, Y. Chen, Q. Liu, Q. Wang, J. J. Richards, D. Tullman-Ercek, K. R. Shull, M. Wang, Quantitative high-throughput measurement of bulk mechanical properties using commonly available equipment. *Mater. Horiz.* **10**, 97–106 (2023).

26. C. A. Tweedie, D. G. Anderson, R. Langer, K. J. Van Vliet, Combinatorial material mechanics: High-throughput polymer synthesis and nanomechanical screening. *Adv. Mater.* **17**, 2599–2604 (2005).

27. J. Hopewell, R. Dvorak, E. Kosior, Plastics recycling: Challenges and opportunities. *Philos. Trans. R. Soc. B* **364**, 2115–2126 (2009).

28. T. Muringayil Joseph, S. Azat, Z. Ahmadi, O. Moini Jazani, A. Esmaeili, E. Kianfar, J. Haponiuk, S. Thomas, Polyethylene terephthalate (PET) recycling: A review. *Case Stud. Chem. Environ. Eng.* **9**, 100673 (2024).

29. R. Demets, K. Van Kets, S. Huysveld, J. Dewulf, S. De Meester, K. Ragaert, Addressing the complex challenge of understanding and quantifying substitutability for recycled plastics. *Resour. Conserv. Recycl.* **174**, 105826 (2021).

30. M. P. Fleury, L. D. D. Nascimento, C. A. Valentin, J. Lins da Silva, M. P. D. Luz, Creep behaviour of recycled poly(ethylene) terephthalate non-woven geotextiles. *Polymers* **13**, 752 (2021).

31. G. R. Koerner, R. M. Koerner, Leachate flow rate behavior through geotextile and soil filters and possible remediation methods. *Geotext. Geomembr.* **11**, 401–430 (1992).

32. H. Y. Jeon, Chemical resistance and transmissivity of nonwoven geotextiles in waste leachate solutions. *Polym. Test.* **25**, 176–180 (2006).

33. A. Kadoma, Q. Jiao, J. J. Vlassak, Z. Suo, Hydrolytic crack growth and embrittlement in poly(ethylene terephthalate). *J. Mech. Phys. Solids* **176**, 105303 (2023).

34. M. Arhant, M. L. Gall, P. Y. L. Gac, Fracture test to accelerate the prediction of polymer embrittlement during aging—Case of PET hydrolysis. *Polym. Degrad. Stab.* **196**, 109848 (2022).

35. M. D. M. C. López, A. I. Ares Pernas, M. J. Abad López, A. L. Latorre, J. M. López Vilariño, M. V. González Rodríguez, Assessing changes on poly(ethylene terephthalate) properties after recycling: Mechanical recycling in laboratory versus postconsumer recycled material. *Mater. Chem. Phys.* **147**, 884–894 (2014).

36. F. Fraternali, V. Ciancia, R. Chechile, G. Rizzano, L. Feo, L. Incarnato, Experimental study of the thermo-mechanical properties of recycled PET fiber-reinforced concrete. *Compos. Struct.* **93**, 2368–2374 (2011).

37. S. Shi, L. Lin, On the environmental stress cracking of a vibration-welded polycarbonate (PC)-based nanocomposite. *Polymer* **285**, 126338 (2023).

38. V. Gritsichine, J. Vlachopoulos, M. R. Thompson, Understanding environmental stress cracking in the Bell Test at the notch region. *Polym. Eng. Sci.* **64**, 264–278 (2023).

39. V. Gritsichine, H. S. Marway, J. Vlachopoulos, M. R. Thompson, Improving the consistency of environmental stress cracking resistance testing for polyethylenes. *Polym. Eng. Sci.* **63**, 478–488 (2022).

40. L. Andena, L. Castellani, A. Castiglioni, A. Mendogni, M. Rink, F. Sacchetti, Determination of environmental stress cracking resistance of polymers: Effects of loading history and testing configuration. *Eng. Fract. Mech.* **101**, 33–46 (2013).

41. A. Samadi-Dooki, M. A. Lamontia, J. D. Londoño, C. Williamson, H. E. Burch, M. Yahyazadehfar, L. A. Carbajal, K. Kourtakis, Effect of chain orientation on coupling of optical and mechanical anisotropies of polymer films. *Coatings* **14**, 764 (2024).

42. A. Ajovalasit, G. Petrucci, M. Scafidi, RGB photoelasticity applied to the analysis of membrane residual stress in glass. *Meas. Sci. Technol.* **23**, 025601 (2012).

43. D. C. Wright, *Environmental Stress Cracking of Plastics* (iSmithers Rapra Publishing, 1996).

44. J. Williams, G. Marshall, Environmental crack and craze growth phenomena in polymers. *Proc. R. Soc. Lond. A Math. Phys. Sci.* **342**, 55–77 (1975).

45. F.-M. Preda, A. Alegría, A. Bocahut, L. A. Fillot, D. R. Long, P. Sotta, Investigation of water diffusion mechanisms in relation to polymer relaxations in polyamides. *Macromolecules* **48**, 5730–5741 (2015).

46. K. Tonyali, C. E. Rogers, H. R. Brown, Stress-cracking of polyethylene in organic liquids. *Polymer* **28**, 1472–1477 (1987).

47. M. Thuy, M. Pedragosa-Rincon, U. Niebergall, H. Oehler, I. Alig, M. Bohning, Environmental stress cracking of high-density polyethylene applying linear elastic fracture mechanics. *Polymers* **14**, 2415 (2022).

48. S. N. Zhurkov, Kinetic concept of the strength of solids. *Int. J. Fract. Mech.* **1**, 311–323 (1965).

49. H. Eyring, Viscosity, plasticity, and diffusion as examples of absolute reaction rates. *J. Chem. Phys.* **4**, 283–291 (1936).

50. T. Smith, C. Gupta, Z. Fan, G. J. Brust, R. Vogelsong, C. Carr, S.-Q. Wang, Toughness arising from inherent strength of polymers. *Extreme Mech. Lett.* **56**, 101819 (2022).

51. T. G. Townsend, *Landfill Bioreactor Design & Operation* (Routledge, 2018).

52. P. Kjeldsen, M. A. Barlaz, A. P. Rooker, A. Baun, A. Ledin, T. H. Christensen, Present and long-term composition of MSW landfill leachate: A review. *Crit. Rev. Environ. Sci. Technol.* **32**, 297–336 (2002).

53. H. Tada, C. P. Paris, R. G. Irwin, *The Stress Analysis of Cracks Handbook* (ASME Press, 2000).

54. M. Shi, Q. Jiao, T. Yin, J. J. Vlassak, Z. Suo, Hydrolysis embrittles poly(lactic acid). *MRS Bull.* **48**, 45–55 (2022).

55. A. Stern, F. Asanger, R. W. Lang, Creep crack growth testing of plastics—II. Data acquisition, data reduction and experimental results. *Polym. Test.* **17**, 423–441 (1998).

56. G. R. Irwin, Analysis of stresses and strains near the end of a crack traversing a plate. *J. Appl. Mech.* **24**, 361–364 (1957).

57. IPCC, *Climate Change 2021: The Physical Science Basis*. Contribution of Working Group I to the Sixth Assessment Report of the Intergovernmental Panel on Climate Change (Cambridge Univ. Press, 2021).

58. G. Wernet, C. Bauer, B. Steubing, J. Reinhard, E. Moreno-Ruiz, B. Weidema, *ecoinvent Database Version 3.12* (ecoinvent Association, 2021).

59. F. Awaja, D. Pavel, Recycling of PET. *Eur. Polym. J.* **41**, 1453–1477 (2005).

60. F. Ronkay, B. Molnár, D. Nagy, G. Szarka, B. Iván, F. Kristály, V. Mertinger, K. Bocz, Melting temperature versus crystallinity: New way for identification and analysis of multiple endotherms of poly(ethylene terephthalate). *J. Polym. Res.* **27**, 372 (2020).

61. T. Bárány, J. Karger-Kocsis, T. Czigány, Effect of hygrothermal aging on the essential work of fracture response of amorphous poly(ethylene terephthalate) sheets. *Polym. Degrad. Stab.* **82**, 271–278 (2003).

62. B. Cotterell, J. Reddel, The essential work of plane stress ductile fracture. *Int. J. Fract.* **13**, 267–277 (1977).

63. A. B. Martínez, N. León, D. Arencón, M. Sánchez-Soto, Essential work of fracture, crack tip opening displacement, and J-integral relationship for a ductile polymer film. *Polym. Test.* **55**, 247–256 (2016).

64. E. A. Patterson, Z. F. Wang, Towards full field automated photoelastic analysis of complex components. *Strain* **27**, 49–53 (1991).

65. J. W. Dally, Dynamic photoelastic studies of fracture. *Exp. Mech.* **19**, 349–361 (1979).

66. L. R. G. Treloar, The photo-elastic properties of rubber. Part I: Theory of the optical properties of strained rubber. *Trans. Faraday Soc.* **43**, 277–284 (1947).

67. J. C. Briñez-de León, H. López-Osorio, M. Rico-García, H. Fandiño-Toro, Deep learning as a powerful tool in digital photoelasticity: Developments, challenges, and implementation. *Opt. Laser. Eng.* **180**, 108274 (2024).

68. Plascene, PET resin tariffs 2025: How U.S. brands can save 10–20% with plascene (2025); https://plascene.com/plascene-com-pet-resin-tariffs-2025/.

69. IMARC Group, Recycled PET (rPET) pricing report (2025); https://imarcgroup.com/recycled-pet-pricing-report/.

70. R. M. Koerner, *Designing with Geosynthetics* (Xlibris Corporation, 2012), vol. 1.

71. Industrial Physics, Multi-sample test system (2025); https://industrialphysics.com/product/multi-sample-test-system/.

72. TA Instruments, Multi-specimen fatigue 16 (2025); https://tainstruments.com/multi-specimen-fatigue-instruments/.

73. K. Huang, C. Kain, N. Diaz-Vallejo, Y. Sohn, L. Zhou, High throughput mechanical testing platform and application in metal additive manufacturing and process optimization. *J. Manuf. Process.* **66**, 494–505 (2021).

74. B. Boyce, B. Salzbrenner, Apparatus for high-throughput sequential tensile testing and methods thereof. U.S. Patent 11,002,649B1 (2021).

75. J. D. McColskey, A. J. Slifka III, E. S. Drexler, M. Dvorak, Multiple specimen testing. U.S. Patent 9,188,519B2 (2015).

76. NASA Technology Transfer, Ultra high-throughput small punch test machine for maximum efficiency (MFS-TOPS-128) (2025); https://technology.nasa.gov/patent/MFS-TOPS-128/.

77. J. J. Schwartz, M. Wood, J. Ye, A. Jaycox, X. Zhong, A. Gongora, High throughput materials screening. U.S. Patent Application 2024/0096454A1 (2024).

78. A. Baker, M. Wong, High throughput mechanical strain generating system for cell cultures and applications thereof. WO Patent WO2014/165056A1 (2014).

79. S. Shahriar, R. Weng, R. Hudson, L. Gotwalt, W. Luo, "Rare-event uncertainty quantification of additively manufactured composites with high-throughput tests," in *AIAA SCITECH 2025 Forum* (American Institute of Aeronautics and Astronautics; 2025), paper 2025–1556.

80. Y. Zhou, S. Lin, X. Zhang, H. Wu, J. Blanchet, Z. Suo, T. Lu, Is a high-throughput experimental dataset large enough to accurately estimate a statistic? *J. Mech. Phys. Solids* **183**, 105521 (2024).

81. H. Wu, X. Zhang, Y. Zhou, J. Blanchet, Z. Suo, T. Lu, Detection and reduction of systematic bias in high-throughput rupture experiments. *J. Mech. Phys. Solids* **174**, 105249 (2023).

82. Y. Zhou, X. Zhang, M. Yang, Y. Pan, Z. Du, J. Blanchet, Z. Suo, T. Lu, High-throughput experiments for rare-event rupture of materials. *Matter* **5**, 654–665 (2022).

83. A. Barrios, C. Kunka, J. Nogan, K. Hattar, B. L. Boyce, Automated high-throughput fatigue testing of freestanding thin films. *Small Methods* **7**, e2201591 (2023).

84. H. J. Kim, J. H. Han, R. Kaiser, K. H. Oh, J. J. Vlassak, High-throughput analysis of thin-film stresses using arrays of micromachined cantilever beams. *Rev. Sci. Instrum.* **79**, 045112 (2008).

85. J. L. Sormana, S. Chattopadhyay, J. Carson Meredith, High-throughput mechanical characterization of free-standing polymer films. *Rev. Sci. Instrum.* **76**, 062214 (2005).

86. L. Zhang, G. Wang, H. Yan, X. Yao, High-throughput characterization of mechanical parameters of thin-film array. *Meas. Sci. Technol.* **33**, 105903 (2022).
